# Pre-hatching embryo-dependent and -independent programming of endometrial function in cattle

**DOI:** 10.1371/journal.pone.0175954

**Published:** 2017-04-19

**Authors:** Mariana Sponchiado, Nathália Souza Gomes, Patrícia Kubo Fontes, Thiago Martins, Maite del Collado, Athos de Assumpção Pastore, Guilherme Pugliesi, Marcelo Fábio Gouveia Nogueira, Mario Binelli

**Affiliations:** 1 School of Veterinary Medicine and Animal Science, University of São Paulo, Pirassununga, São Paulo, Brazil; 2 Department of Pharmacology, São Paulo State University, Botucatu, São Paulo, Brazil; 3 Department of Veterinary Medicine, Faculty of Animal Science and Food Engineering, University of São Paulo, Pirassununga, São Paulo, Brazil; 4 Androvet, Sertãozinho, São Paulo, Brazil; 5 Department of Clinic and Surgery of Veterinary, School of Veterinary, Minas Gerais Federal University, Belo Horizonte, Minas Gerais, Brazil; 6 Department of Biological Science, São Paulo State University, Assis, São Paulo, Brazil; Universite du Quebec a Trois-Rivieres, CANADA

## Abstract

The bovine pre-implantation embryo secretes bioactive molecules from early development stages, but effects on endometrial function are reported to start only after elongation. Here, we interrogated spatially defined regions of the endometrium transcriptome for responses to a day 7 embryo *in vivo*. We hypothesize that exposure to an embryo changes the abundance of specific transcripts in the cranial region of the pregnant uterine horn. Endometrium was collected from the uterotubal junction (UTJ), anterior (IA), medial (IM) and posterior (IP) regions of the uterine horn ipsilateral to the CL 7 days after estrus from sham-inseminated (Con) or artificially inseminated, confirmed pregnant (Preg) cows. Abundance of 86 transcripts was evaluated by qPCR using a microfluidic platform. Abundance of 12 transcripts was modulated in the Preg endometrium, including classical interferon-stimulated genes (*ISG15*, *MX1*, *MX2* and *OAS1Y*), prostaglandin biosynthesis genes (*PTGES*, *HPGD* and *AKR1C4*), water channel (*AQP4*) and a solute transporter (*SLC1A4*) and this was in the UTJ and IA mainly. Additionally, for 71 transcripts, abundance varied according to region of the reproductive tract. Regulation included downregulation of genes associated with proliferation (*IGF1*, *IGF2*, *IGF1R* and *IGF2R*) and extracellular matrix remodeling (*MMP14*, *MMP19* and *MMP2*) and upregulation of anti-adhesive genes (*MUC1*) in the cranial regions of uterine horn. Physical proximity to the embryo provides paracrine regulation of endometrial function. Embryo-independent regulation of the endometrial transcriptome may support subsequent stages of embryo development, such as elongation and implantation. We speculate that successful early embryo-dependent and -independent programming fine-tune endometrial functions that are important for maintenance of pregnancy in cattle.

## Introduction

In cattle, pre-implantation embryo development starts after successful fertilization and continues until initial migration of giant trophoblast cells from the conceptus trophectoderm to the maternal luminal epithelium and lasts approximately 20 days [1]. The morula-stage embryo reaches the uterus 4 to 5 days post-estrus, develops to the blastocyst stage by day 7, hatches from the zona pellucida on days 9–10 and develops into a tubular conceptus that begins to elongate on day 15 to a filamentous form that occupies the entire length of the ipsilateral uterine horn by day 19 [2]. Around day 16 the apposition and transient attachment of the trophoblastic cells to the uterine epithelium begins. After day 19, the elongating conceptus is adhered to the luminal epithelium and placentation starts [3].

An important feature of the pre-implantation embryo development in cattle is that the embryo/conceptus relies solely on uterine secretions (i.e., the histotroph) to supply required nutrients and growth factors. The histotroph is composed of molecules synthesized and secreted by the endometrial glandular and luminal epithelia as well as selectively transported from blood [4]. Endometrial secretion and transport of molecules to the uterine lumen are spatially and temporally programmed processes. Programming can be put forth by embryo-independent and -dependent factors and their interaction.

Fluctuations of sex steroid concentrations during the periovulatory period and throughout pre-implantation development exert classical endocrine, embryo-independent programming, while secretions from the developing embryo/conceptus act on a paracrine fashion inside the uterine lumen to modulate endometrial function. Dysregulation of this complex interplay leads to early embryonic mortality, which ranges from 25 to 30% in beef cattle [5].

Regarding embryo/conceptus-mediated programming of endometrial function, a critical unanswered question is when in pre-implantation development does it start [6]. It is well established that after starting elongation, there is an increasing capacity of the conceptus to secrete interferon-tau (IFNT; [7]), which modulates prostaglandin synthesis in the endometrium to block luteolytic pulses of prostaglandin F2α (PGF_2α_) and to favor prostaglandin E2 (PGE_2_) secretion [8, 9]. However, to the best of our knowledge, there is no evidence that the embryo affects endometrial function before elongation, which begins 13 days after AI [10]. However, *in vitro* culture studies demonstrated that preimplantation embryos secrete a range of biochemical messengers that act in concert to promote embryonic development, referred to as embryotropins (reviewed by Wydooghe et al. [11]). Interestingly, many of these factors have cognate receptors expressed in the uterus. Activation of such receptors could lead to cellular and tissue responses such as transcription and *de novo* synthesis of proteins and metabolites, as well as post-transcriptional and post-translational modifications of molecules pre-existing in the endometrium [12]. It is reasonable to expect that pre-elongation embryo-derived factors regulate endometrial transcription in regions that are in close physical association to the embryo. In the present study we interrogated spatially defined regions of the endometrium transcriptome for responses to a day 7 embryo *in vivo*. We hypothesize that exposure to an embryo changes the abundance of specific transcripts in the cranial region of the pregnant uterine horn.

Regarding sex-steroid programming of endometrial function, (1) manipulation of pre-ovulatory follicle growth and associated changes in proestrus estradiol (E2) and diestrus progesterone (P4) concentrations [13–15] and (2) exogenous supplementation of P4 during early diestrus regulates the endometrial transcriptome and function [16, 17], and fertility [18]. More importantly, the effects of sex steroid hormones depend on their bioavailability to the endometrium and the nature and abundance of specific receptors in the endometrium. Regarding bioavailability, anatomical evidence indicates a distinct sex-steroids input according to the region of the reproductive tract. Specifically, the vascular arrangement of vessels that irrigate the uterus allows a greater input of ovarian steroids to the cranial portion of the uterine horn ipsilateral to the ovary containing the CL compared to the cranial portion of the contralateral horn and compared to the mid-caudal region of either horn [19]. Araújo et al. [20] proposed that such spatial changes in ovarian steroid input regulate local endometrium gene expression and function. Furthermore, they showed decreasing endometrial abundance of *PGR* and *ESR2* transcripts from the cranial to the caudal region of the uterine horn. This suggests that responsiveness to steroids may also be regionally controlled. Despite the clear implications of such regional specificities on the regulation of uterine function to support conceptus development, there is a lack of information on target pathways that could be modulated along the uterine horn.

Objective was to measure pre-hatching embryo-dependent and -independent effects on the abundance of select transcripts associated with uterine function to support gestation along the uterine horn in beef cows.

## Materials and methods

Experiments were carried out at the University of São Paulo in Pirassununga, São Paulo, Brazil. All experimental procedures involving animals were approved by the Ethics and Animal Handling Committee of the School of Veterinary Medicine and Animal Science of the University of São Paulo (CEUA-FMVZ/USP, n3167260815). Protocol was in accordance with the ethical principles in animal research.

### Reproductive management and treatments

All animals were maintained in a single *Brachiaria brizantha* pasture, supplemented with chopped sugarcane, concentrate and minerals to fulfill their maintenance requirements and received water *ad libitum*.

**Fig 1:**
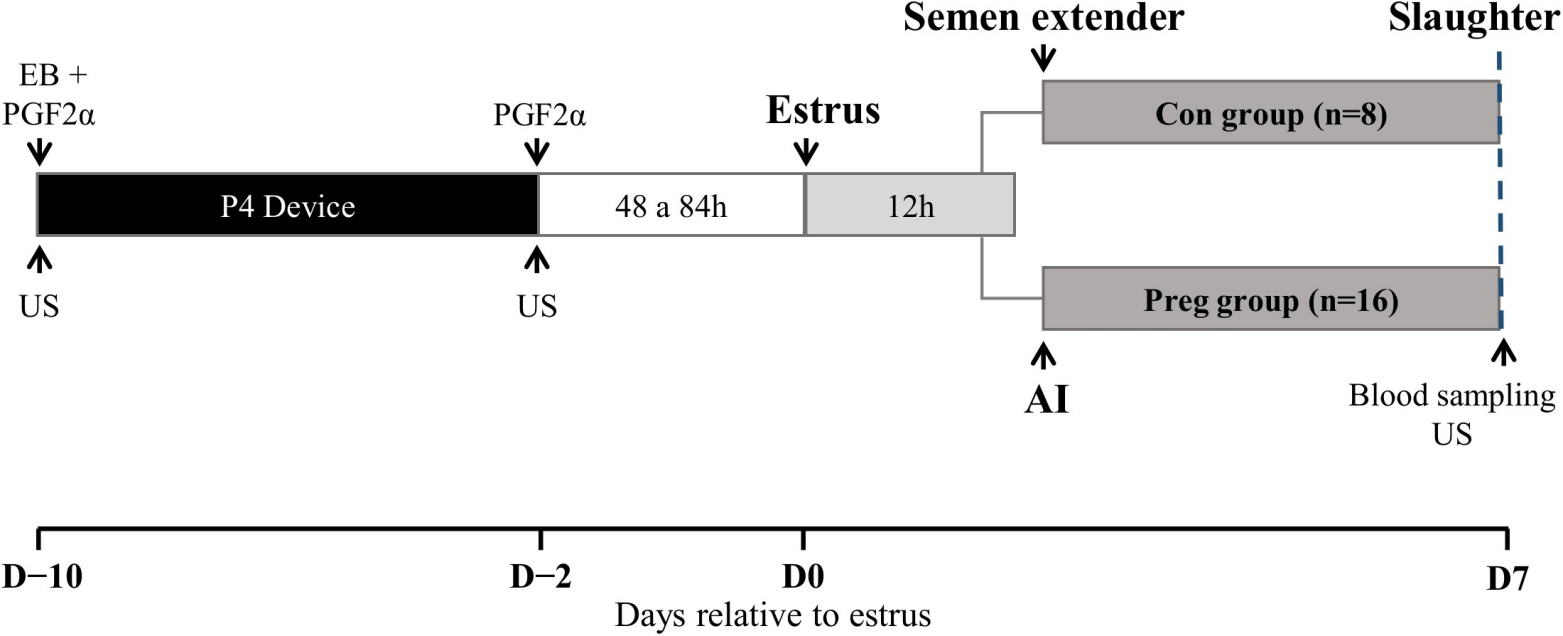
Experimental design. The estrous cycles of Nelore cows (n = 36) were synchronized using an 8-day progesterone-releasing intravaginal device. On day –10 (D–10), cows received a progesterone-releasing device (1 g; Sincrogest; Ourofino) and an injection of 2 mg estradiol benzoate (EB; Sincrodiol, Ourofino) and an injection of prostaglandin F2α (PGF2α; 500 μg of sodium cloprostenol; Sincrocio, Ourofino). On D–3, when the progesterone-releasing device was removed, all cows received an extra injection of PGF2α. At estrus (D0), cows were allocated to one of two experimental groups: Control (Con), cows were sham-inseminated and received semen extender; Pregnant (Preg), cows were inseminated with semen from the same batch of semen from a bull of proven fertility, 12 h post-estrus. All cows were slaughtered on D7.

The estrous cycles of reproductively normal multiparous Nelore (*Bos taurus indicus*, n = 36; average body weight 531 ± 12 kg) cows were synchronized by insertion of an intravaginal P4-releasing device (1 g; Sincrogest®, Ourofino Saúde Animal, Cravinhos, São Paulo, Brazil) and i.m. administrations of PGF_2α_ analogue (500 μg of sodium cloprostenol; Sincrocio®, Ourofino Saúde Animal) and estradiol benzoate (2 mg; Sincrodiol®, Ourofino Saúde Animal) on day –10 (D–10; Fig 1). At the time of P4-device removal (D–3), animals received an i.m. administration of PGF_2α_ and an Estrotect^TM^ heat detector patch (Rockway, Inc. Spring Valley, WI, USA). Cows were visually observed for signs of estrus activity twice a day between 48 and 84 h after P4-releasing device withdrawal. Cows observed in standing heat or presenting an activated heat detector patch were considered in estrus (n = 30; D0 of the study). Animals were allocated randomly to one of two experimental groups 12 h after standing estrus. In the control group (Con; n = 8), cows were sham-inseminated with deposition of semen extender in the uterine body; in the Pregnant group (Preg; n = 16), cows were artificially inseminated with frozen-thawed semen from the same batch of a bull of proven fertility. All procedures were performed by a single technician. Ovulation was checked 12 hours later by B-mode transrectal ultrasonography, and only cows with a confirmed, single ovulation were maintained in the experiment (n = 24).

Transrectal ultrasonography (7.5-MHz transducer, DP-50 vet; Mindray, Shenzhen, Guangdong, China) was also performed to measure CLs and follicles on D–10 and D–3, side and size of the preovulatory follicle and ovulation on D0 and D1, and to confirm the CL development on D7.

### Endometrial sample collection

**Fig 2:**
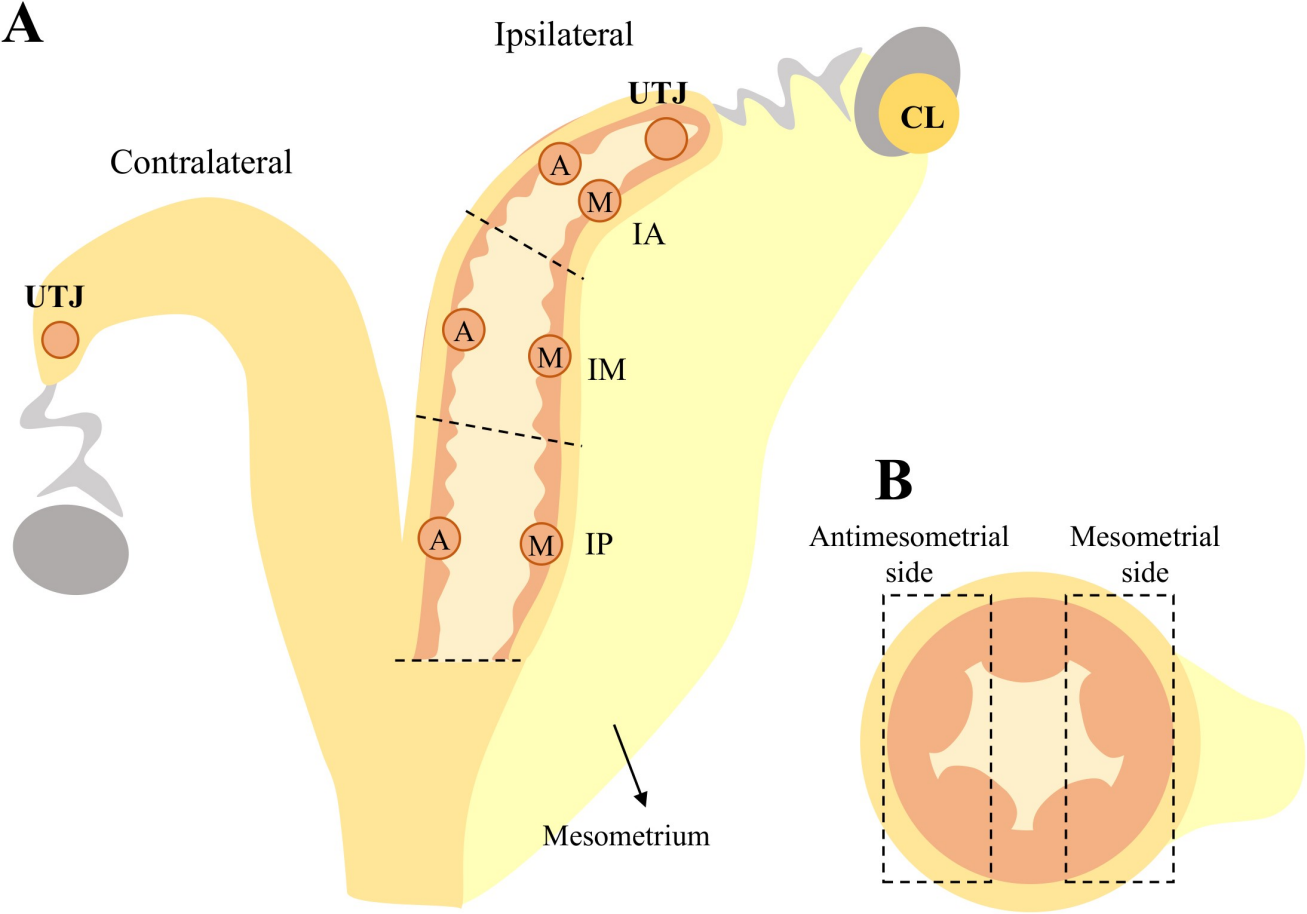
Schematic representation of sites selected for collection of endometrial samples. Panel A: Endometrial tissue samples were collected from 8 regions of the uterus: uterotubal junction (UTJ) ipsi and contralateral to the ovary containing the CL and the intermediate portion of the anterior, medial and posterior thirds of the ipsilateral uterine horn, that were further divided in mesometrial (M) and antimesometrial (A) sides. Panel B: Representative cross-section of the uterine horn indicating the sites for endometrium collection according to the mesometrium insertion.

Animals were slaughtered on D7 after estrus. Between 4 and 8 animals, from both experimental groups, were slaughtered in each of four independent sessions. Reproductive tracts were isolated and transported on ice to the laboratory within 10 min to uterus processing. Uterus were trimmed free of surrounding tissues. The ipsi and contralateral horns relative to ovary containing the CL were isolated. Average uterine horns length was 27.12 ± 0.70 cm [mean ± SEM]. For each uterine horn, always starting from the horn ipsilateral to the CL, forceps were placed every 8 cm starting from the utero-tubal junction (UTJ) to delimit the anterior, medial and posterior uterine thirds (Fig 2). The anterior, medial and posterior thirds were individually washed with 3, 5 and 6 mL of PBS, respectively, and the flushing was recovered in a petri dish. The presence and location of an embryo in the flushings was verified under a stereomicroscope in the Preg group animals. All embryos found (n = 10/16) were at the expected stage of development (compact morula or early, not-hatched blastocyst) and were in the flushing obtained from the anterior third. Inseminated cows from which no embryo was recovered (n = 6) were excluded from the experiment.

After flushing, the ipsilateral uterine thirds were incised longitudinally at the mesometrial insertion. From each third, a 1 cm-wide strip of intercaruncular endometrium was dissected transversally from the lengthwise intermediate portion of the third. Then, each strip was subdivided in mesometrial and antimesometrial sides. Endometrial component of the ipsi and contralateral UTJ was collected, but not further subdivided. Once collected, the endometrial samples were immediately transferred to cryotubes and snap frozen in liquid nitrogen. Samples were stored at –80°C until further processing. Only intercaruncular endometrium was collected because there are no caruncules in the UTJ region. In addition, only intercaruncular endometrium contains endometrial glands, which are functional units that play a major role on histotroph secretion.

### Blood sampling and progesterone concentration measurements

Prior to slaughter, blood samples were collected via jugular venipuncture for subsequent measurement of P4. Blood samples were collected into 10 mL heparinized evacuated tubes (BD, São Paulo, SP, Brazil) and were maintained in ice for 1 hour until plasma separation. Samples were centrifuged at 4°C, 1,500 x g for 15 min and plasma was extracted and stored in sterile 2 mL vials at –20°C until assayed.

Plasma P4 concentrations were measured by solid-phase radioimmunoassay (Immuchem Double Antibody Progesterone Kit, MP Biomedicals, Germany GmbH, Eschwege) according to manufacturer’s instructions. The intra-assay coefficients of variation were 0.17% (low concentration reference) and 7.39% (high concentration reference). The detection limit (sensibility) of the assay was 0.1 ng/mL.

### RNA extraction and quality analysis

Endometrial fragments (~40mg) were mechanically macerated in liquid nitrogen using a stainless steel apparatus. Subsequently, the macerate was homogenized in lysis buffer from PureLink^®^ RNA mini kit (Ambion^TM^, Life Technologies, Carlsbad, California, USA) and further RNA extraction performed as per manufacturer’s instructions. To maximize lysis, tissue suspension was passed at least ten times through a 21-ga needle, and centrifuged at 12,000 g for 1 min at 4°C for removal of cellular debris. Supernatant was loaded and processing in RNeasy columns. Total RNA was eluted with 30 μL of RNase free water. Concentration and purity of total RNA in extracts were evaluated using spectrophotometry (NanoVue^TM^ Plus Spectrophotometer, GE Healthcare, UK) by the absorbance at 260 nm and the 260/280 nm ratios, respectively.

RNA integrity was assessed using automated capillary gel electrophoresis on a Bioanalyzer 2100 with RNA 6000 Nano Lab-chips (Agilent Technologies Ireland, Dublin, Ireland) according to manufacturer’s instructions. Absorbance ratios (28S/18S) and RNA integrity values recorded for all RNA samples extracted ranged between 1.8 and 2.0, and 6.9 and 9.8, respectively.

Samples of RNA (200 ng) were treated for contaminating genomic DNA using DNase I Amplification Grade (Invitrogen^TM^, Life Technologies) in accordance with manufacturer’s guidelines supplied. First strand cDNA was synthesized using the High Capacity cDNA Reverse Transcription kit (Applied Biosystems™, Life Technologies) with RNaseOUT Recombinant Ribonuclease Inhibitor (Invitrogen™, Life Technologies) according to manufacturer’s instructions. Total RNA was reverse transcribed using random hexamers and incubated at 25°C for 10 min, followed by incubation at 37°C for 2 h and reverse-transcriptase inactivation at 85°C for 5 min. The cDNA was stored at −20°C for subsequent analyses.

### Primers pairs selection and validation

Transcript abundance was determined by microfluidic dynamic array using BioMark HD (Fluidigm, South San Francisco, CA, USA) platform in a 96.96 Dynamic Array™ Integrated Fluidic Circuits (Fluidigm), which enables reaction of 96 cDNA samples with 96 genes assays in a single run. Representative genes were selected from 11 key pathways known to influence endometrial function, in addition to endogenous controls (Table 1). Primer details are provided on Supplemental S1 Table.

**Table 1:** Representative genes selected from key pathways known to influence endometrial function and endogenous controls.

**Cell-cell adhesion**	**Eicosanoid metabolic process**	**Growth factor signaling**	**Secretory activity**	**Solute and water transport**	**Sex steroid signaling**
*FN1* *ICAM1* *ICAM3* *ITFG3* *ITGAV* *ITGB1* *LGALS1* *LGALS7B* *LGALS9* *MUC1* *VIL1*	*AKR1B1* *AKR1C4* *HPGD* *PTGES* *PTGES2* *PTGES3* *PTGIS* *PTGS1* *PTGS2* *SLCO2A1*	*EDN3* *EGFR* *FGF2* *FGFR2* *FLT1* *GRB7* *IGF1* *IGF1R* *IGF2* *IGF2R* *IGFBP7* *KDR*	*GRP* *LTF* *MCOLN3* *PIP* *RBP4* *SCAMP1* *SCAMP2* *SCAMP3* *SERPINA14* *SPP1*	*AQP1* *AQP4* *CLDN10* *SLC13A5* *SLC1A4* *SLC2A1* *SLC5A6* *SLC7A8*	*ESR1* *ESR2* *GPER* *OXTR* *PAQR8* *PGR1* *PGRMC1* *PGRMC2*
**Interferon Signaling**	**Extracellular matrix assembly**	**Extracellular matrix remodeling**	**Oxidative Stress**	**Polyamine Regulation and proteolysis**	**Endogenous control**
*IFI6* *IFNAR2* *IRF6* *ISG15* *MX1* *MX2* *OAS1Y*	*HAS3* *HMMR* *HYAL1* *HYAL2*	*MMP14* *MMP19* *MMP2* *TIMP2* *TIMP3*	*CAT* *GPX4* *SOD1* *SOD2*	*AMD1* *ODC1* *ANPEP* *EED*	*ACTB* *GAPDH* *PPIA*

Optimized primer pairs were designed using the Primer Express 3.0 based on GenBank Ref-Seq mRNA sequences of target genes. Oligos were aligned by Primer-BLAST (http://www.ncbi.nlm.nih.gov/tools/primer-blast/), to verify their identity and homology to the bovine genome. Oligonucleotides were commercially synthesized as purified salt-free products by Invitrogen (Life Technologies, São Paulo, SP, Brazil). All primer pairs were tested for their sensitivity and specificity first in conventional Real Time qPCR analysis to verify amplification conditions. Briefly, reactions were carried out in 96-well plates sealed with MicroAmp optical adhesive cover (Life Technologies) using the Step One Plus apparatus (Applied Biosystems Real-Time PCR System; Life Technologies). Reactions were conducted in a final volume of 20 μL using 10 μL of Power SYBR Green PCR Master Mix (Life Technologies). The PCR program consisted of an initial denaturation step at 95°C for 10 min, followed by 40 cycles of 15 seconds at 95°C and annealing at 60°C for 1 min. Melting curves were obtained by stepwise increases in the temperature from 60 to 95°C. Primer validation consisted of meeting the following criteria: i) a melt curve containing a sharp peak and devoid of additional peak(s), ii) efficiency of the standard curve ranging between 85% and 115% (based on the slope calculated for 3-fold serial dilution of a pooled cDNA sample), and iii) no amplification of the negative control (diethyl pyrocarbonate treated water replacing template cDNA on the qPCR reaction). The qPCR products identities were confirmed by sequencing and agarose gel electrophoresis for all target genes.

### Transcript abundance analyzes

Transcript abundance analysis in endometrial samples was performed using pre-selected bovine-specific primers. The mRNA abundance of 86 genes was analyzed, as indicated in Table 1, according to functional categories. Prior to qPCR thermal cycling, each sample was submitted to sequence-specific pre-amplification process as follows: 0.5 μL assay (bovine-specific primer forward and reverse, final concentration of 500nM), 2.5 μL 2X TaqMan^®^ PreAmp Master Mix (Applied Biosystems, Foster City, CA, USA), 0.75 μL DNase-free water and 1.25 μL cDNA. The reactions were activated at 95°C for 10 min followed by denaturing at 95°C for 15 s, and annealing and amplification at 60°C for 4 min for 10 cycles. After thermal-cycling, the pre-amplification reactions products were submitted to the process of clean up with Exonuclease I (New England BioLabs, Ipswich, MA, New England): 1.4 μL DNase-free water, 0.2 μL Exonuclease I Reaction Buffer and 0.4 μL Exonuclease I, 20 U/μL, the digest phase was performed by 30 minutes at 37°C, followed by inactivate phase at 80°C for 15 minutes. The final product was diluted 7-fold prior to qPCR analysis. For gene expression analysis, the sample solution prepared consisted of 2.25 μL cDNA (pre-amplified products), 2.5 μL of SsoFast EvaGreen Supermix with low ROX (Bio-Rad, Hercules, CA, EUA) and 0.25 μL of 20X DNA Binding Dye (Fluidigm); and the assay solution: 0.25 μL of 100 μM combined forward and reverse primers, 2.25 μL of 1X DNA Suspension Buffer and 2.5 μL of 2X Assay Loading Reagent (Fluidigm). The 96.96 Dynamic Array™ Integrated Fluidic Circuits (Fluidigm) chip was used to data collection. After priming, the chip was loaded with 5 μL of each assay solution and 5 μL of each sample solution. The qPCR thermal cycling was performed in the Biomark HD System (Fluidigm) running 25 cycles using the protocol GE Fast 96x96 PCR+Melt. A negative control (diethyl pyrocarbonate treated water) was included and a primer pair (*GAPDH*) was essayed in duplicate. Quantitative analysis was carried out by using the crossing point (Cq) values during the log-linear phase of the reaction at which fluorescence increased above background for each primer assay.

Analysis of putative reference genes for qPCR studies was carried out using GeNorm version 3.5 Microsoft Excel Add in (Microsoft, Redmond, WA; [21]). The stability of the transcript abundance of reference genes including peptidylprolyl isomerase A (*PPIA*), actin beta (*ACTB*) and glyceraldehyde-3-phosphate dehydrogenase (*GAPDH*) was investigated across all samples in this study. Relative abundance was obtained after normalization of the target genes Cq values by the geometric mean of *PPIA*, *GAPDH* and *ACTB* transcript abundance values.

### Statistical analysis

Data were analyzed using the Statistical Analysis Systems software package (SAS Inst. Inc., Cary, NC, USA) version 9.3. Continuous data were tested with Shapiro-Wilk and Levene’s test to check the normality of residues and homogeneity of variances, respectively. Group effect (Con *vs*. Preg) was determined by one-way ANOVA using Type III sums of squares.

Transcript abundance data were analyzed by repeated measures in space using the MIXED procedure in two distinct models. The first model estimated the effects of group and uterine regions by Split-plot ANOVA. Fixed effects included the main plot: experimental groups (Con *vs*. Preg); sub-plot: regions (UTJ *vs*. IA *vs*. IM *vs*. IP) and the group by region interaction. The experimental unit was a plot with its unique combination of experimental group and region. Animal within treatment was used as the error term. The type of variance-covariance structure was chosen according to the magnitude of the Akaike information criterion corrected (AICc). The matrix with the least AICc value to each variable was deem best. When the effect of a categorical variable was significant, the Pdiff *post-hoc* test was used to determine differences between means. In the case of a significant interaction, the slice command was incorporated to the procedure to measure group effects within each region. In these analyzes, the mean relative abundance of a transcript on the antimesometrial and mesometrial sides within each uterine region was calculated to represent the region on comparisons.

The second repeated-measures analyzes aimed to compare transcript abundance between groups, uterine regions and sides, by Split-split-plot ANOVA. Fixed effects included the main plot: experimental groups (Con *vs*. Preg); sub-plot: regions (IA *vs*. IM *vs*. IP); sub-sub-plot: sides (Antimesometrial *vs*. Mesometrial) and the resulting double and triple interactions. The experimental unit was a plot with its unique combination of experimental group, region and side. Animal within treatment was used as the error term. The criteria to select the variance-covariance structure and means test were the same as described above.

Pearson’s coefficient of correlation was calculated by CORR procedure. All data are expressed as means ± standard error of the mean (±SEM). Treatment differences with *P* ≤ 0.05 were considered significant and probability of 0.05 < *P* ≤ 0.10 were considered to approach significance.

### Cluster analysis by region

Transformed group means were used for K-means clustering by Euclidian distances using the multivariate tool in Minitab statistical software (Minitab Inc., State College, PA, USA) version 17.1.0. Dendrogram was used for preliminary assessments of the number of gene clusters. Genes with significant group by region interaction were excluded from this analysis.

## Results

### Animal model, ovarian and endocrine variables

Hormonal synchronization successfully generated groups of animals presenting similar ovarian morphologies and sex steroid endocrine profiles (Table 2), as expected. Specifically, POF diameter and CL area, plasma P4 concentration and diameter of the largest follicle on D7 were similar between groups (*P* > 0.1). In the present study, plasma E2 concentrations were not quantified, but the similar largest follicle diameter measured both prior to ovulation and on D7 indicated that groups experienced similar exposure to E2 both during pre-ovulatory period and early diestrus.

**Table 2:** Size of ovarian structures and P4 concentrations in pregnant (Preg) and sham inseminated (Con) cows.

	Group		
Variables	Con (n = 8)	Preg (n = 10)	*P-*value
Pre-ovulatory follicle diameter (mm)	13.57 ± 0.71	13.87 ± 0.41	0.70
CL area on D7 (cm^2^)	2.84 ± 0.24	2.69 ± 0.17	0.62
Plasma P4 concentrations on D7 (ng/mL)	3.67 ± 0.52	3.43 ± 0.45	0.72
Largest follicle diameter on D7 (mm)	11.90 ± 0.45	11.56 ± 0.51	0.68

Values are expressed as means ± SEM.

### Confirmation of transcript abundance data generated by the microfluidic dynamic array method

To measure the repeatability of measurements taken by the Biomark HD system, the same primer pair for a reference gene (*GAPDH*) was ran twice in the same assay for all samples. The correlation coefficient (r) of this test was r = 0.99. To validate transcript abundance results measured by the Biomark microfluidic dynamic array system, abundance of *PPIA*, *IRF6*, *MX2* and *ISG15* transcripts from the same samples were measured by conventional Real time PCR. For each primer pair the correlation between Cqs obtained from Step One Plus and BioMark HD PCR analysis was 0.89, 0.94, 0.93 and 0.89 for *PPIA*, *IRF6*, *MX2* and *ISG15*, respectively.

### Pre-hatching embryo modulation of endometrial transcript abundance

**Fig 3:**
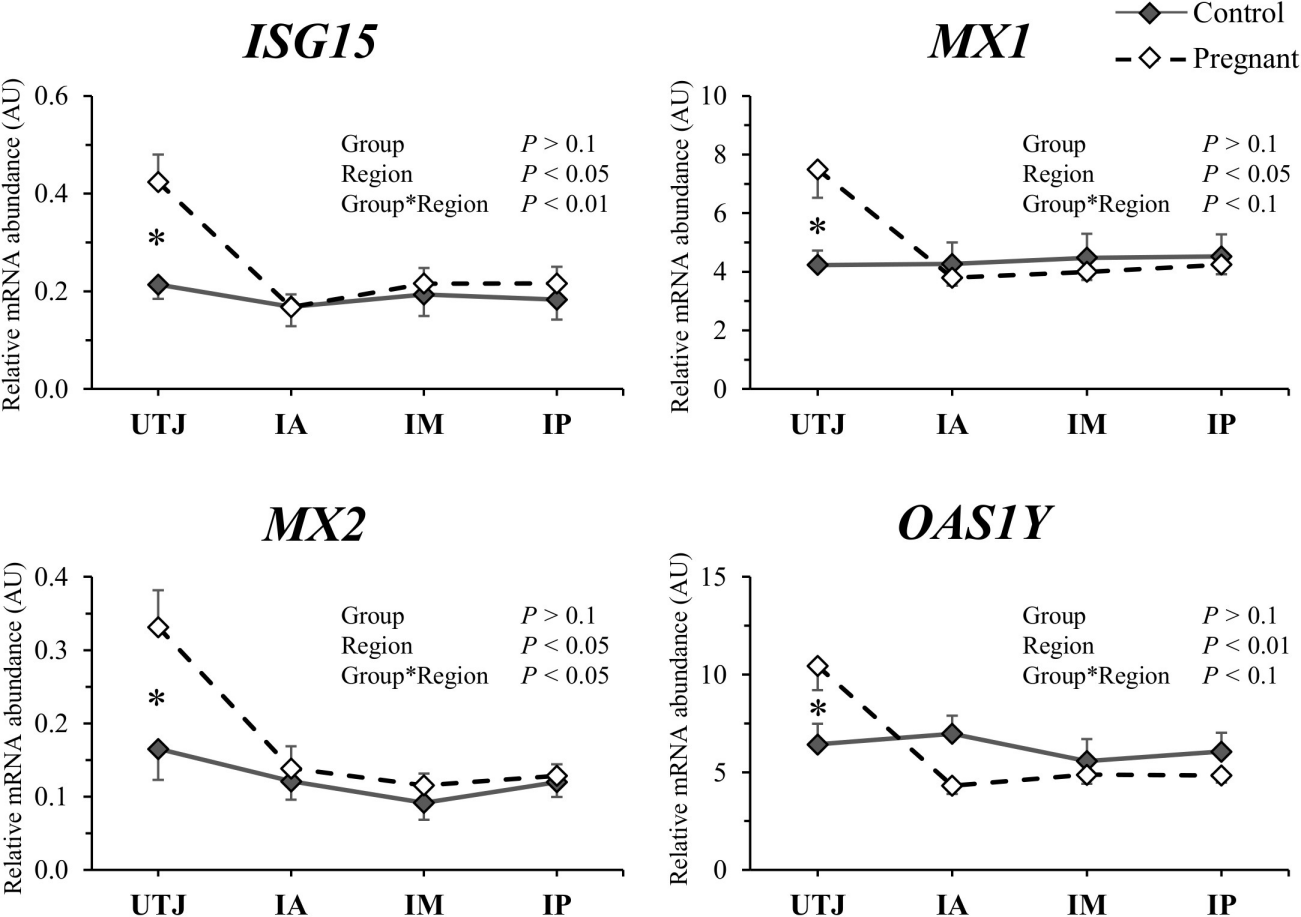
Relative mRNA abundance of interferon-stimulated genes (arbitrary units; AU; mean ± SEM) for control (solid lines) and pregnant (dashed lines) cows in the uterotubal junction (UTJ), anterior (IA), medial (IM) and posterior (IP) regions of the uterine horn ipsilateral to the CL. *P < 0.05 and ˟*P* < 0.1 denotes that significant differences were reached or approached, respectively, between groups at each specific region.

**Fig 4:**
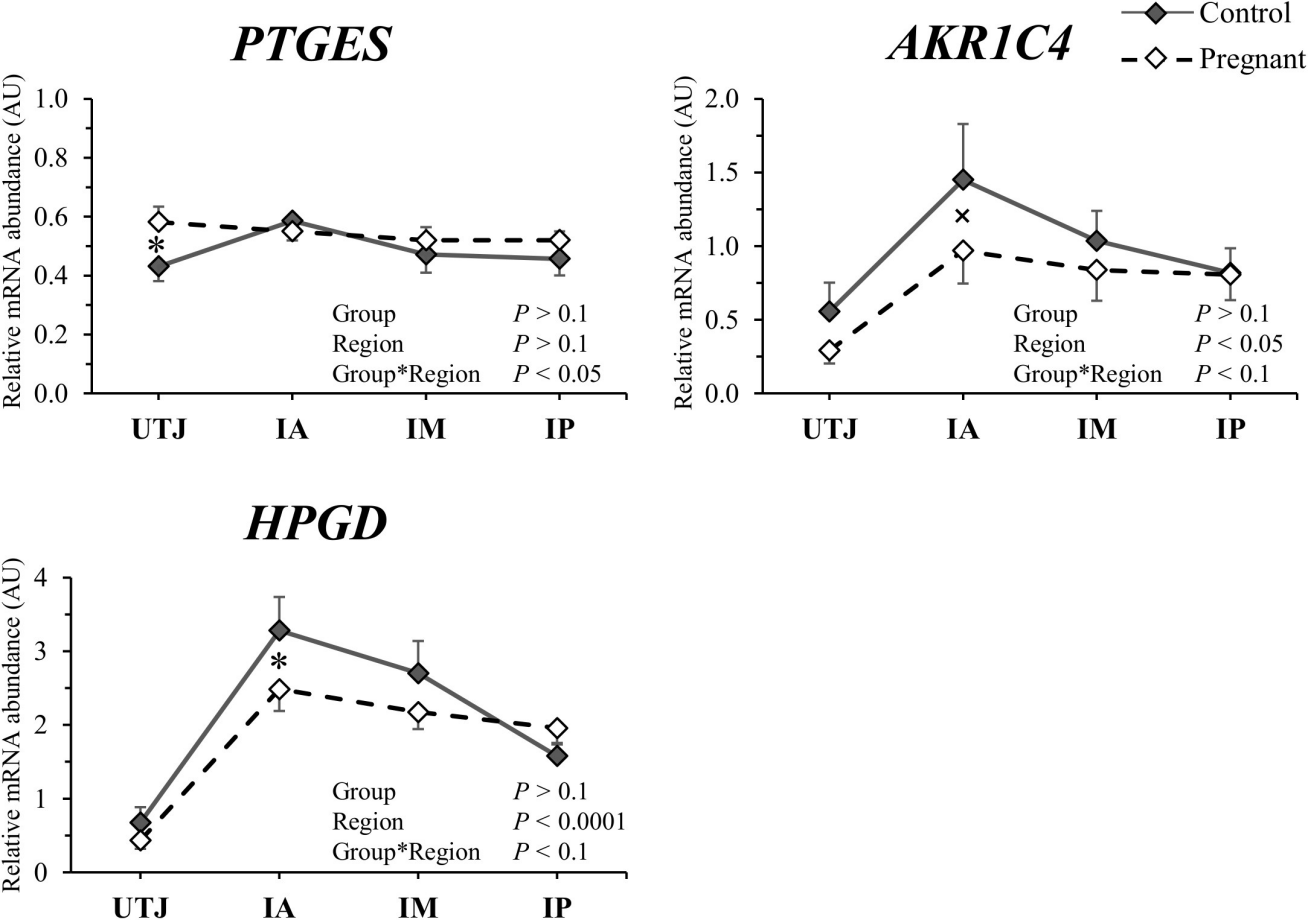
Relative mRNA abundance of eicosanoid biosynthesis related genes (arbitrary units; AU; mean ± SEM) for control (solid lines) and pregnant (dashed lines) cows in the uterotubal junction (UTJ), anterior (IA), medial (IM) and posterior (IP) regions of the uterine horn ipsilateral to the CL. *P < 0.05 and ˟*P* < 0.1 denotes that significant differences were reached or approached, respectively, between groups at each specific region.

**Fig 5:**
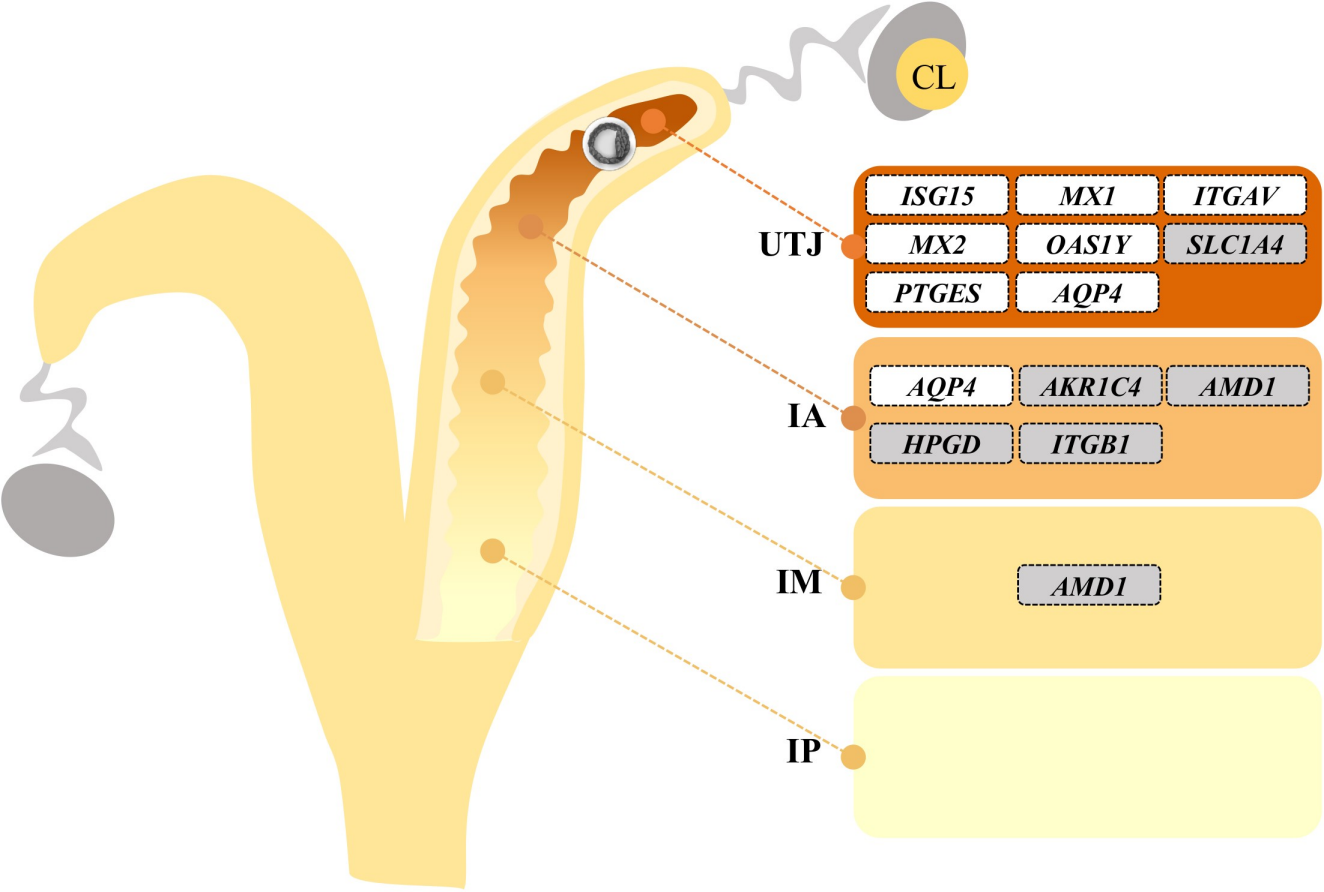
Summary of embryo-dependent effects. Schematic representation of distribution of transcripts affected by Group by Region (UTJ, uterotubal junction; IA, anterior; IM, medial; IP, posterior) interaction in the uterine horn ipsilateral to the CL. The position of each differentially expressed gene in the figure indicate an upregulation (placed inside a white box) or downregulation (placed inside a gray box) in the Pregnant compared to Control cows within each region.

There was no main effect of group (Con *vs*. Preg) for the abundance of any of the transcripts measured; but there was a significant group by region (UTJ, IA, IM or IP) interaction for 12 of the 83 transcripts (Table 3). Interpretation of these interactions revealed that the group effect manifested predominantly in the regions in which the embryos were found (i.e. UTJ and anterior third). Remarkably, four differentially expressed genes were classical interferon stimulated genes. The abundances of *ISG15*, *MX1*, *MX2* and *OAS1Y* mRNAs were respectively 1.98, 1.77, 2.00 and 1.50-fold greater in the UTJ of Preg *vs*. Con cows, but abundances were similar between groups in the remaining regions (Fig 3). These results suggest that early embryo-derived IFNT locally induced the expression of ISGs in the endometrium. Furthermore, three differentially expressed genes were related to eicosanoid biosynthesis. The abundance of *PTGES* (a PGE_2_ synthase) in the UTJ from Preg cows was upregulated (1.35-fold). Further, in the IA region from Preg group the abundance of *AKR1C4* (a PGF_2α_ synthase) and *HPGD* (an enzyme involved in prostaglandin catabolism) transcripts was reduced 0.67 and 0.76-fold, respectively (Fig 4). Such expression patterns are consistent with the early embryo-mediated induction of PGE_2_ and inhibition of PGF_2α_ synthesis in the endometrium. The proportion of transcript abundance between Preg and Con groups for the remaining genes differentially expressed (*P* ≤ 0.1) in the UTJ were *AQP* (1.58-fold), *ITGAV* (1.30-fold) and *SLC1A4* (0.52-fold). Transcripts abundance for *AMD1* (0.72-fold), *APQ4* (1.64-fold) and *ITGB1* (0.83-fold) differed (*P* < 0.05) between groups in the IA region. Only *AMD1* mRNA differed (0.77-fold; *P* < 0.05) in the IM region. A summary of embryo-dependent regional modulation of endometrial transcript abundance is presented in Fig 5.

**Table 3:** Effects of Group (Preg *vs*. Con), Region (UTJ, IA, IM *vs*. IP) and Group by Region in the abundance of transcripts showing significant Group by Region (G*R) interaction.

Gene symbol	Overall effects *P*-value			Ratio of mean transcript abundance (Preg:Con)^a^^,^^b^			
	Group	Region	G*R	UTJ	IA	IM	IP
Interferon Signaling							
*ISG15*	ns^c^	0.04	0.02	1.98*	1.00	1.12	1.18
*MX1*	ns	0.02	0.06	1.77*	0.89	0.89	0.94
*MX2*	ns	0.05	0.03	2.00*	1.15	1.26	1.07
*OAS1Y*	ns	0.01	0.08	1.50 ˟	0.77	0.76	0.80
Eicosanoid metabolic process							
*AKR1C4*	ns	0.02	0.07	0.52	0.67˟	0.81	0.99
*HPGD*	ns	0.00	0.08	0.64	0.76*	0.80	1.24
*PTGES*	ns	ns	0.04	1.35*	0.94	1.10	1.14
Cell-cell adhesion							
*ITGAV*	ns	ns	0.07	1.30˟	0.87	1.05	1.17
*ITGB1*	ns	0.00	0.04	1.03	0.83*	0.91	1.04
Polyamine Regulation							
*AMD1*	ns	0.00	0.04	0.98	0.72**	0.77*	0.99
Solute and water transport							
*AQP4*	ns	0.01	0.07	1.58*	1.64*	1.06	0.98
*SLC1A4*	ns	ns	0.09	0.52*	1.11	0.72	0.93

Within each region, the abundance of each transcript was compared between Pregnant (Preg) and Control (Con) groups.

^a^Magnitude of effect of group (Preg *vs*. Con) within each region is indicated by: **P ≤ 0.01; *P ≤ 0.05; ˟*P* ≤ 0.1.

^b^Data are presented as the ratio of mean transcript abundance between Preg and Con groups

^c^Not significant (P > 0.1).

To confirm that the up regulation of ISGs abundance in Preg UTJ was not caused by the presence or passage of sperm, we compared the abundances of *ISG15*, *MX1* and *MX2* transcripts in the UTJ from the uterine horn contralateral to the CL between Preg (contacted sperm but not the embryo) and Con (did not contact sperm or embryo) groups. Abundance of ISGs was similar between groups for all genes tested *(P* > 0.1; data in S1 Fig) in the contralateral horns. Therefore, group effect noted in the ipsilateral horn was due the passage or presence of the embryo, which was unique to the ipsilateral horn and not sperm, which contacted both uterine horns.

### Pre-hatching embryo-independent regional regulation of endometrial transcript abundance

**Fig 6:**
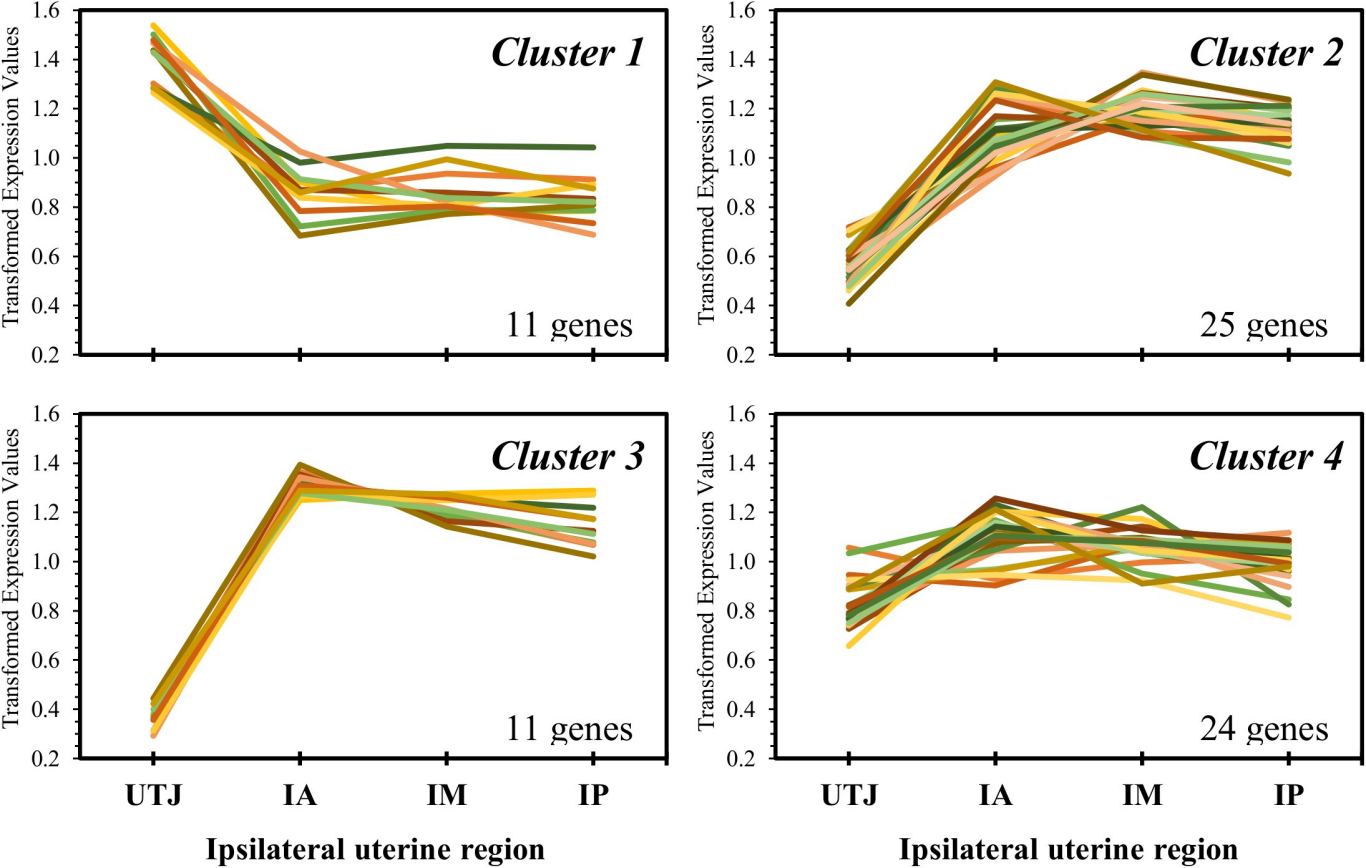
Cluster analysis showing uterine spatial signatures of transcript abundance in the uterotubal junction (UTJ), anterior (IA), medial (IM) and posterior (IP) regions of the ipsilateral uterine horn. Relative expression was mean-centered and clustered by K-means. Genes present in each cluster are in Table 4.

We evaluated transcript abundance of 83 key genes associated with endometrial receptivity to the embryo in 4 regions along the ipsilateral uterine horn and established a spatial signature of endometrial transcript abundance. There was an effect of region in the expression pattern of most genes (85.5% [71/83]; Supplemental S2 Table). Genes with similar abundance patterns along the ipsilateral uterine horn were grouped by K-means analysis in four clusters. Regional patterns of expression are represented in Fig 6; a list of gene symbols assigned to each cluster is in Table 4. In general, we observed a contrasting pattern of transcript abundance in the UTJ compared to the other regions. It is remarkable that in the UTJ region there were many genes that were downregulated compared to the remaining regions, in which the expression patterns were more balanced (Clusters 2 and 3). Specifically, Cluster 1 presents a strikingly different pattern of expression in comparison to Clusters 2 and 3; there are 11 genes upregulated in the UTJ region, which are associated with developing embryo support, (*FGF2*, *PTGIS* and *SLCO2A1*), interferon signaling (*IFNAR2* and *IFI6*) and adhesive glycoproteins (*FN1* and *MUC1*). Cluster 2 was composed of 25 genes that were downregulated in the UTJ and whose expression increased gradually in the IA, IM and IP thirds. Interestingly, some of these genes are associated with a non-receptive endometrium. For example, *IGF1*, *IGF1R*, *IGF2*, *IGF2R*, and *KDR* are associated with proliferation, and *MMP14*, *MMP19*, *MMP2* and *TIMP3* are linked to extracellular matrix remodeling. The Cluster 3 included 11 genes whose expression was drastically downregulated in the UTJ and similar among others regions, including *AKR1B1*, *ANPEP*, *SLC13A5* and *SLC5A6*. Finally, the genes in cluster 4 (24 genes) presented similar expression pattern across the four uterine regions.

**Table 4:** Genes representing clusters shown in Fig 6.

Cluster	Gene Symbols
1	*FGF2*, *FGFR2*, *FN1*, *GRP*, *IFI6*, *IFNAR2*, *MUC1*, *OXTR*, *PTGIS*, *PTGS1*, *SLCO2A1*
2	*EED*, *FLT1*, *GPX4*, *HMMR*, *HYAL1*, *HYAL2*, *ICAM1*, *IGF1*, *IGF1R*, *IGF2*, *IGF2R*, *ITFG3*, *KDR*, *LGALS7B*, *MMP14*, *MMP19*, *MMP2*, *PGR1*, *PGRMC1*, *PTGES2*, *RBP4*, *SCAMP3*, *SLC7A8*, *TIMP3*, *VIL1*
3	*AKR1B1*, *ANPEP*, *EDN3*, *ESR2*, *GRB7*, *IRF6*, *MCOLN3*, *ODC1*, *PAQR8*, *SLC13A5*, *SLC5A6*
4	*AQP1*, *CAT*, *CLDN10*, *EGFR*, *ESR1*, *GPER*, *HAS3*, *ICAM3*, *IGFBP7*, *LGALS1*, *LGALS9*, *LTF*, *PGRMC2*, *PIP*, *PTGES3*, *PTGS2*, *SCAMP1*, *SCAMP2*, *SERPINA14*, *SLC2A1*, *SOD1*, *SOD2*, *SPP1*, *TIMP2*

### Pre-hatching embryo-independent side regulation of endometrial transcript abundance

**Fig 7:**
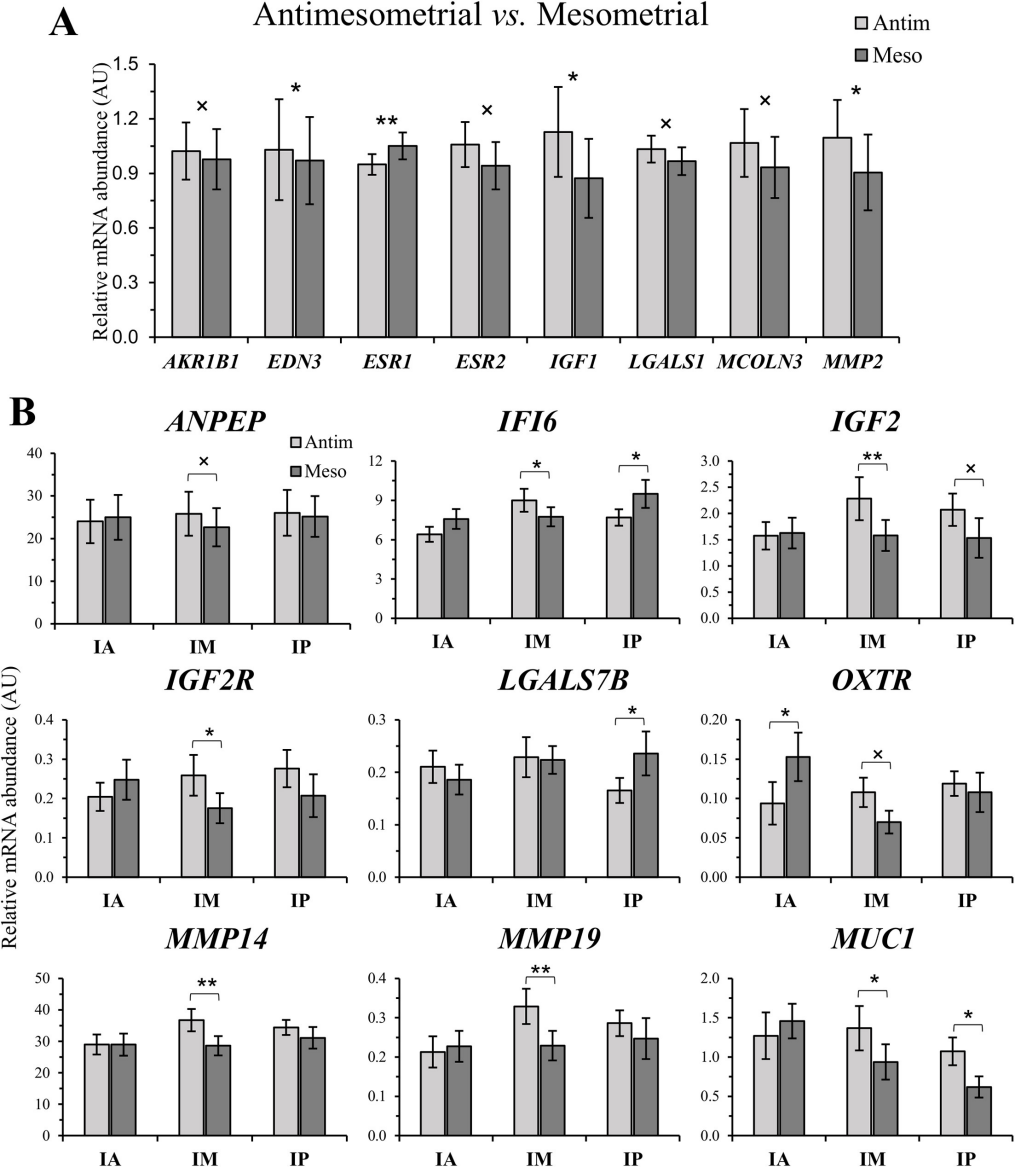
Relative mRNA abundance of genes affected by Side (antimesometrial *vs*. mesometrial; Panel A; arbitrary units: AU; mean ± SEM) and by the Side by Region (IA, IM *vs*. IP; Panel B; arbitrary units: AU; mean ± SEM) interaction in the uterine horn ipsilateral to the CL. *P < 0.05 and ˟*P* < 0.1 denotes that significant differences were reached or approached, respectively, between sides at each specific region.

**Fig 8:**
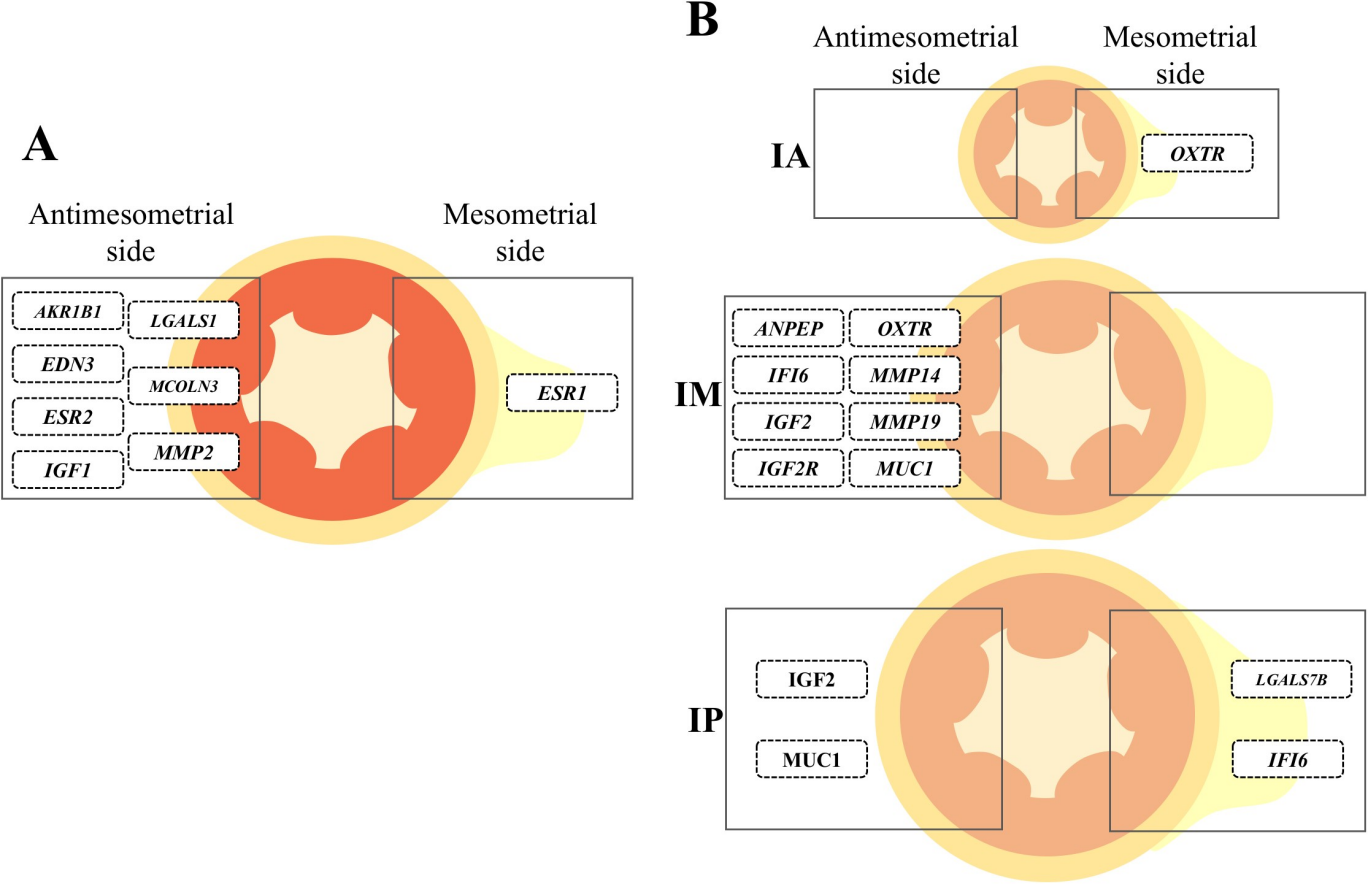
Summary of side-dependent differences in gene expression. Schematic representation of distribution of genes affected by Side (antimesometrial *vs*. mesometrial; Panel A) and by the Side by Region (IA, anterior; IM, medial; IP, posterior; Panel B) interaction in the uterine horn ipsilateral to the CL. The position of each differentially expressed gene in the figure indicate an upregulation in the side.

Transcript abundance in the mesometrial and antimesometrial sides of the uterine horn was evaluated on IA, IM and IP uterine thirds. In general, the effect of side was slight. From the 83 genes evaluated, the abundance of only eight genes (~10% [8/83]) was affected by side (Fig 7A) and the abundance of nine genes (~11% [9/83]) was affected by a region by side interaction (Fig 7B). These analyzes revealed that the main effect of side was predominant in the IM and IP uterine thirds. In general, transcript abundance was less in the mesometrial side, indicating the presence of inhibitory effects in proximity to larger vessel blood supply. A summary of side-dependent modulation of endometrial transcript abundance is presented in Fig 8.

## Discussion

A critical unanswered question surrounding early pregnancy in cattle is how early is the embryo able to regulate endometrial function to favor its development. Studies over the past 20 years have indicated the existence of complex paracrine and endocrine *in vivo* communication between early embryo and the maternal tract in mammalians [22, 23]. *In vitro* culture studies demonstrated that preimplantation embryos secrete a range of biochemical messengers that act in concert to promote embryonic development, referred to as embryotropins (reviewed by Wydooghe et al. [11]). The aim of the present study was to compare the abundance of specific transcripts between cycling and pregnant bovine endometria to elucidate if presence of a pre-hatching embryo might influence the endometrial function at a time-point coinciding with apical uterine position of the embryo. We demonstrated for the first time that a day 7 embryo was able to locally regulate interferon signaling and eicosanoid biosynthesis pathways in the endometrium. Furthermore, embryo-independent, uterine region and side regulation of transcript abundance was discovered.

The pre-hatching, day 7 embryo is located in the cranial portion of the uterine horn ipsilateral to the CL, that comprehended the UTJ and cranial-most 8 cm of the horn. In agreement, Dinskin & Sreenan [5] reported that embryos recovered 8 days post-estrus were located at the tip of the uterine horn, close to the UTJ, in inseminated beef cows. The dynamic of migration of the early bovine embryo along the uterine horn remains poorly known. According to Wolf et al. [24] the embryo does not float in a recognizable volume of maternal secretions, but is surrounded tightly by the endometrium through a thin fluid layer stabilized by glycoproteins.

Exposure to a day 7 embryo stimulates local expression of classic interferon-induced genes. The fact that the abundance of transcripts for ISGs (*ISG15*, *MX1*, *MX2* and *OAS1Y*) was increased in the UTJ region of Preg cows is suggestive of signaling by embryo secreted interferon-tau. Furthermore, there was a greater abundance of transcripts for *IFNAR2*, a classical interferon type I receptor, in the UTJ than other uterine regions. This could be associated with a more pronounced interferon-mediated response in this region. The expression of *IFNT* mRNA and protein is first evident as the trophoblast cell lineage develops at the late morula and early blastocyst stage in cattle (D6–7 of pregnancy; [25, 26]). *In vivo* or *in vitro* derived day 7 bovine blastocysts produce very low amounts of IFNT (~ 100 to 1000 pM/day) as measured by antiviral cell protection assay [7]. Indeed, *in vitro* stimulation of endometrial cells with 25 nmol of IFNT was needed to increased *ISG15* mRNA and ISG15 protein abundance [27]. Thus, it is remarkable that such early embryos were capable to change endometrial transcript abundance as reported here. Probably, such limited capacity of synthesis and secretion of signals by the early embryo [28] is the reason of the locally restricted effects verified. Substantial endometrial expression of ISGs was reported previously [9] in embryo recipients on D13 after estrus.

To rule out the possibility of non-specific ISGs stimulation by exposure to sperm in Preg cows, we compared ISG expression between Con and Preg cows in the contralateral UTJ. The similar ISG transcript abundance between the groups further indicated that the significant differences found in the ipsilateral UTJ were induced by the embryo. Thus, although IFNT signaling is likely to have occurred in the present study, functional relevance of such early communication is currently unknown.

Exposure to a day 7 embryo changes the abundance of eicosanoid metabolism transcripts to favor a greater PGE_2_/PGF_2a_ ratio. Prostaglandins evidently regulate endometrial functions and conceptus elongation during early pregnancy [8]. Dorniak et al. [29] showed that intrauterine infusion of meloxican, a selective PTGS2 inhibitor, prevented conceptus elongation in early pregnant sheep. In the present report, the expression of *PTGES* (prostaglandin E synthase) was upregulated, while that of *AKR1C4* (aldo-keto reductase family 1, member C4) and *HPGD* (hydroxyprostaglandin dehydrogenase 15-(NAD)) was downregulated in pregnant endometria. The PTGES enzyme converts PGH_2_ to PGE_2_ and is mainly responsible for the production of the PGE_2_ in the bovine endometrium [30]. The AKR1C4 enzyme converts PGH_2_ to PGF_2α_, while HPGD is responsible for prostaglandins catabolism [31]. Interpretation of these data suggests an increase in PGE_2_ synthesis and secretion and decrease in PGF_2α_ synthesis, which is a pro-gestation phenotype. Indeed, PGE_2_ has been associated to multiple roles as an embryo and luteotrophic signal and as an important mediator in endometrial receptivity, myometrial quiescence, and immune function at the fetal-maternal interface during the establishment of pregnancy [8, 32, 33]. Conversely, uterine production of PGF_2α_ has a direct negative effect on continued embryonic development [34]. Seals et al. [35] verified that most susceptible period of embryonic growth to the negative effects of PGF_2α_ was during the development from morula to blastocyst, which happens at the apical uterine portion. Consistent with our findings, Beltman et al. [36] analyzed the tip of the uterus and verified that the expression of *PTGES* was upregulated in the endometria of heifers with a viable embryo compared to that of a retarded embryo, while the expression of *HGPD* was significantly decreased in this group.

Remaining transcripts regulated by the embryo locally were *ITGB1* (in the IA region) and *AMD1* (in the IA and IM regions), both downregulated in the Preg group, and *AQP4* (in the UTJ and IA regions), upregulated by the embryo. The integrin subunit beta 1 (ITGB1) is a glycoprotein involved in the cell-cell adhesion, cell-extracellular matrix adhesion and signal transduction, and is expressed along the basolateral membranes of the luminal and glandular epithelial cells as well as around the blood capillaries throughout the endometrium [37]. Guillomot [38] provide evidence that major components of the ECM and the ITGB1 are lost in a progressive local pattern during the trophoblastic adhesion process in the caprine endometrium. The adenosylmethionine decarboxylase 1 is an enzyme coded by *AMD1* gene and is implicated in polyamine biosynthesis. The biological relevance of this finding during early pregnancy has not been established. However, Heald [39] observed that local embryonic signals seems to regulate polyamine synthesis in the pregnant uterus of rats. The embryo-induced regulation of *AQP4* transcripts indicates an increased water transport to the portion of the horn containing the embryo. The aqueous transport through the aquaporin channel is driven by osmotic gradients. Secretion, absorption and homeostasis of uterine fluid are crucial for embryo development [40].

Regional differences in transcript abundance along the uterine horn ipsilateral to the CL define a functional spatial signature associated with receptivity to the embryo. Cluster analysis grouped four patterns of transcript abundance along the ipsilateral uterine horn. Cluster 1 represents 11 genes that showed overexpression in the UTJ region and that support the developing embryo, such as *FGF2*, *FGFR2*, *PTGIS* and *SLCO2A1*, are associated with interferon response (*IFNAR2* and *IFI6*) and provide embryo adhesiveness (*FN1* and *MUC1*). FGF2 has been described as a strong mediator of IFNT production in bovine trophectoderm cells and blastocyst-stage bovine embryos [41] and greater amounts of *FGF2* mRNA in the ipsilateral apical uterine horn can be a reasonable explanation to support the early embryo development. Excess mucin, coded by the *MUC1* gene, prevents embryo attachment to the endometrial luminal epithelium [42, 43]. Thus, upregulation of this gene in the Preg UTJ probably stimulates embryo transit to the subsequent regions of the uterine horn.

Cluster 2 was composed of 25 transcripts whose abundance was lowest in the UTJ and continuously increased in the IA, IM and IP regions. Interestingly, some of these transcripts are associated with a non-receptive endometrium, that expresses proliferation- (*IGF1*, *IGF1R*, *IGF2*, *IGF2R*, and *KDR*) rather than secretion-associated genes [13] and extracellular matrix remodeling genes (*MMP14*, *MMP19*, *MMP2* and *TIMP3;* [15]). The gradual uterine cranial-wise downregulation of these genes may be related to local requirements of the embryo, that are specific to each stage of development. The UTJ-IA location of embryos in the present report are consistent with the concept that an endometrium that is less engaged in proliferation and remodeling is receptive compatible with early embryo requirements. Findings were similar to those described by Bauersachs et al. [44], which identified differential mRNA expression between different regions (anterior, middle and posterior) of the ipsi and contralateral uterine horns. Specifically, that study showed an increase in *UTMP* (also known as *SERPINA14*) transcripts abundance at the cranial ipsilateral uterine horn, similarly to our study. Regulation of region specific transcript profiles may be exerted through differential vascularization along the uterine horn. Specifically, there is a preferential input of blood draining the ovaries to the cranial region of the uterus compared to the mid and posterior regions [19]. Thus, sex steroid regulation of endometrial transcription may explain regional differences in transcript abundance [20].

A collective finding that was consistent to clusters 1 to 3 relates to the uniqueness of the transcription profile in the UTJ compared to the remaining regions of the uterus. To the best of our knowledge, there are no studies comparing the gene expression of UTJ with the remaining uterine horn. The bovine UTJ is composed of three parts: terminal isthmic segment, transition region proper and uterine apex [45]. The uterine apex extends to the point of the first caruncles, approximately 1–1.5 cm caudally to the oviductal transition into the uterine horn. Only the endometrial component of the UTJ was collected and analyzed in this study. The luminal epithelium of the bovine UTJ consists of a simple columnar epithelium containing ciliated and non-ciliated cells, and its surface is covered by varying amounts of a mucous secretion that tends to agglomerate the cilia and microvilli [45]. The existence of glands in the bovine UTJ remains controversial [46], although we clearely detected glands in histologic sections of the uterus (data not shown). Discrepant differences verified at transcriptional level may be due to differences in cellular compartments between regions.

Side differences in transcript abundance along the uterine horn ipsilateral to the CL define a second layer of functional spatial signature associated with receptivity to the embryo. Because the main blood vessels supplying the bovine uterus are inserted through the mesometrium, we hypothesized that there would be a greater input of systemic factors and ovarian steroids in the endometrium close to the mesometrium insertion [47]. Perhaps this could evoke side-specific regulation of gene expression. Furthermore, to the best of our knowledge, there are no reports showing evidence of differential gene expression between mesometrial and antimesometrial sides in the bovine endometrium during pre-hatching embryo development. In the present study, abundance of eight transcripts was affected by side, seven of which were upregulated in the antimesometrial side. One such gene is endothelin 3 (*EDN3*), which is a potent vasoconstrictor. The EDN3 is synthetized by endometrial stromal and glandular epithelial cells and acts in a paracrine manner in the uterine vasculature [48, 49]. A decrease in *EDN3* mRNA abundance in the mesometrial side of endometrium may point to a local vasodilatation and, thus, increased blood supply in this region. Study of region by side interactions revealed additional 9 genes (Fig 8B) whose transcript abundance was regulated between sides in least one uterine region (IA, IM or IP). Interpretation of interactions showed that the majority of side effects are concentrated in the IM and IP regions in comparison to the IA. It is possible that because the caudal-wise increase in the uterine horn diameter, the distance between the mesometrial and antimesometrial sides are greater and regulation is more prone to occur. In many rodents, including mice and rats, attachment always occurs at the antimesometrial side of the uterine lumen, opposite the entry site of blood vessels into the uterus, whereas implantation is mesometrial in bats, mare and pigs [50]. However, relevance of side-specific transcript abundance in cattle is unknown and remains to be discovered. Although not examined in the present study, there are probably embryo-dependent and -independent effects on caruncular endometrium function. Such effects were reported earlier before [51] and at implantation [52].

The functional relevance of uterine programming during pre-hatching early embryo development can be questioned due to the fact that *in vitro* produced bovine embryos can be caudally transferred to the uterus and are able to establish gestations successfully. However, it has been demonstrated clearly in embryo transfer programs that there is a greater pregnancy success when an embryo was transferred deep in ipsilateral uterine horn compared to a shallow transfer (65.9% *vs*. 29.6%; [53]). Furthermore, Newcomb et al. [54] transferred single embryos surgically, bilaterally on day 7 to a combination of sites (tip or base) of uterine horns in cows. They concluded that the tip of the ipsilateral uterine horn is the optimal site for fetal survival and that to ensure a high twin fetal survival one embryo must had been placed in this site. Collectively, these findings and ours provide evidence that although the sequential exposure of the embryo to a regionally programmed uterus is not absolutely required to establishment of pregnancy, absence of exposure may be implied as a contributing factor to reduced establishment of pregnancy, such as when embryo is transferred caudally in cattle. Thus, exposure to the pre-hatching embryo may fine-tune endometrial function to support subsequent pregnancy events.

**Fig 9:**
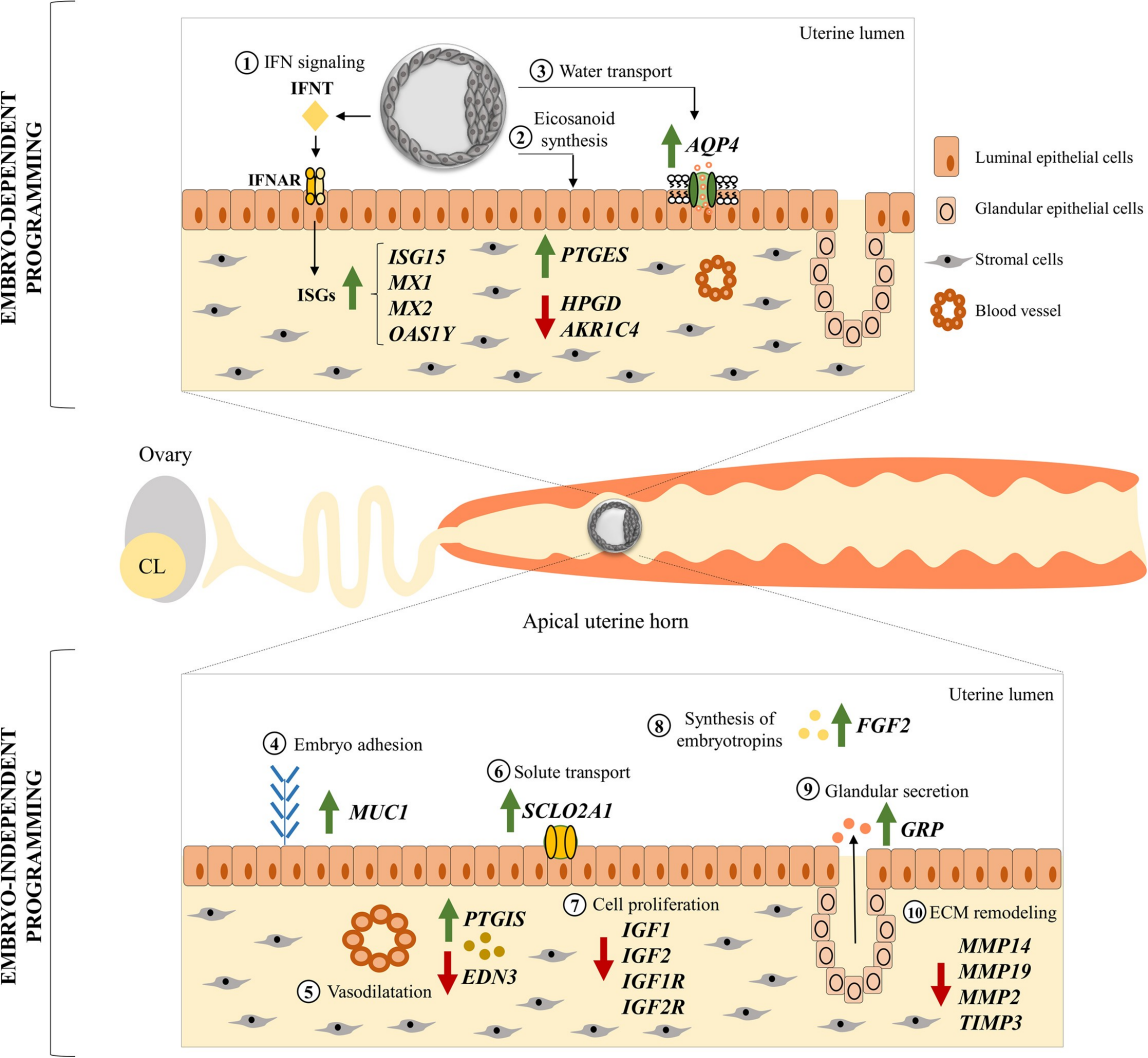
A working model integrating embryo-dependent (top panel) and -independent (bottom panel) programming of endometrial function 7 days after estrus. Numbers 1 to 9 represent processes and associated key genes whose transcription was modulated in the UTJ and/or apical portion of the uterine horn compared to the medial and posterior portions. Changes in transcript abundance are irrespective of cell type, since they were measured on whole endometrium homogenates. *Embryo-dependent signaling*: (1) Interferon-tau (IFNT) secreted by the embryo affects the endometrium in a paracrine manner, regulating the transcription of interferon-stimulated genes (*ISG15*, *MX1*, *MX2* and *OAS1Y*); (2) embryo-induced regulation of eicosanoid metabolism genes favor a greater PGE2:PGF2α ratio; (3) embryo-induced regulation of *AQP4* transcripts indicates an increased water transport to the portion of the horn containing the embryo. *Embryo-independent signaling*: (4) upregulation of the anti-adhesive *MUC1* stimulates transit of the embryo from the UTJ to the more caudal regions of the uterine horn; (5) upregulation of vasodilatation-related gene (*PTGIS*) and downregulation of vasoconstriction-related gene (*EDN3*) indicate greater blood flow to the apical regions of the horn; (6) increase of solute transport (*SLCO2A1*); (7) downregulation of genes associated with cellular proliferation (*IGF1*, *IGF2*, *IGF1R* and *IGF2R*), suggests a change in tissue function to promote (8) synthesis of embryotropins (*FGF2*) and (9) secretion, which is supported by upregulation of glandular secretions-related gene (*GRP*); (10) decrease of extracellular matrix remodeling (*MMP14*, *MMP19*, *MMP2* and *TIMP3*). Collectively, at the transcriptional level, changes are consistent with an endometrial phenotype that is more receptive to the embryo in the apical portion of the uterine horn compared to the medial and posterior portions.

In conclusion, the present study showed that the expression pattern of specific genes in the endometrium respond to pre-hatching embryo-dependent and -independent programming (Fig 9). Embryo-dependent programming requires physical proximity to the embryo probably because of the limited capacity of synthesis and secretion of signals by the early embryo. Clear regional and side changes in transcript abundance were observed in this study and their critical role for further embryo development and survival and, ultimately, pregnancy success, deserves further research. Mechanisms that regulate regional expression of transcripts have not been elucidated, but may include vascular specializations to deliver different sex-steroid concentrations to particular regions of the reproductive tract and specific intrinsic regional programming of expression across the uterine horn. We propose that successful pre-hatching embryo-dependent and -independent programming of endometrial function fine-tune endometrial functions that are important for a successful pregnancy in cattle.

## Supporting information

S1 Table Bovine specific oligonucleotide forward and reverse primer sequences (5’-3’) and PCR product length. (DOCX)

S2 Table Summary of regional effects in the transcripts abundance measured by Real Time PCR in the uterotubal junction (UTJ), anterior (IA), medial (IM) and posterior (IP) samples of the ipsilateral uterine horn. (DOCX)

S1 Fig Relative mRNA abundance of interferon-stimulated genes (arbitrary units; AU; mean ± SEM) for control (white bars) and pregnant (black bars) cows in the uterotubal junction of the uterine horn contralateral to the CL. No significant mean differences were detected (*P* > 0.1).
(TIF)
