# Supplementary material for: Pre-hatching embryo-dependent and -independent programming of endometrial function in cattle
Source: PLoS One. 2017 Apr 19;12(4):e0175954. doi: 10.1371/journal.pone.0175954 (PMC5397052; doi:10.1371/journal.pone.0175954)
Supplement: S1 Table — (DOCX) [file pone.0175954.s001.docx]

**S1 Table.** **Bovine specific oligonucleotide forward and reverse primer sequences (5’-3’) and PCR product length.** (Continued)

| **Gene symbol** | **Gene** | | **GenBank number** | **Forward and Reverse sequences** | | | **Amplicon** | | **Reference** |
| --- | --- | --- | --- | --- | --- | --- | --- | --- | --- |
| **Cell-cell adhesion** | |  | | |  |  | |  | |
| *FN1* | fibronectin 1 | | NM_001163778.1 | 5’ AGTACACAGTCAGTGTGGTTGCCT  3’ AAACTTCAGGTTGGTTGGTGCAGG | | | 101 | | Primer Express |
| *ICAM1* | intercellular adhesion molecule 1 | | NM_174348.2 | 5’ AGACCCTGAAGTGCGAGGCT  3’ TATTCTGGCCGTGGAGCACGTT | | | 198 | | Primer Express |
| *ICAM3* | intercellular adhesion molecule 3 | | NM_174349.1 | 5’ GAACCCGGTCACTATCAACATC  3’ CTTGGCGTCAGGTGGTAAAT | | | 124 | | Primer Express |
| *ITFG3* | family with sequence similarity 234 member A | | NM_001075318.1 | 5’ TGTGGAGGATCGATTACAATGC  3’ CAGCACAGGAGAAGCTGGAAT | | | 139 | | Primer Express |
| *ITGAV* | integrin subunit alpha V | | NM_174367.1 | 5’ TTTCAGGAGTTCCAAGAGCAGCGA  3’ TGAAGAGAGGTGCGCCGATAAACA | | | 183 | | Primer Express |
| *ITGB1* | integrin subunit beta 1 | | NM_174368.3 | 5’ TCAGACTTCCGAATTGGGTTTG  3’ AAATGGGCTCGTGCAGTTCT | | | 118 | | Primer Express |
| *LGALS1* | lectin, galactoside-binding, soluble, 1 | | NM_175782.1 | 5’ TCGTGGAGGTATGCATCTCCTT  3’ TGAAGTCACCACCTGCAGACA | | | 127 | | Primer Express |
| *LGALS7B* | lectin, galactoside-binding, soluble, 7B | | XM_002694977.3 | 5’ GCTTTAACGTCCCCCACAAG  3’ CACCGCACAGCAGGTTCA | | | 118 | | Primer Express |
| *LGALS9* | lectin, galactoside-binding, soluble, 9 | | NM_001039177.2 | 5’ AGGCGGGAACAGGTTTGC  3’ CCTCTCTGCTTCGTGTTGCA | | | 118 | | Primer Express |
| *MUC1* | mucin 1 | | NM_174115.2 | 5’ CAACCAGGGCAATGAGATAG  3’ ACCATCAGCGGAGTTAGT | | | 143 | | Primer Express |
| *VIL1* | villin 1 | | NM_001013591.1 | 5’ GCTGCTCTACACCTACTTCATC  3’ GATCTGGACCGGTTCATTGT | | | 146 | | Primer Express |
| **Eicosanoid metabolic process** | |  | | |  |  | |  | |
| *AKR1B1* | aldo-keto reductase family 1, member B1 | | NM_001012519.1 | 5’ ATACAAGCCGGCGGTTAAC  3’ TGTCTGCAATCGCTTTGATC | | | 188 | | Oliveira et al., 2015^[[1]](#footnote-1)^ |
| *AKR1C4* | aldo-keto reductase family 1, member C4 | | NM_181027.2 | 5’ TCCTGTCCTGGGATTTGGAACCTT  3’ ATCGGCAATCTTGCTTCGAATGGC | | | 166 | | Oliveira et al., 2015^1^ |
| *HPGD* | hydroxyprostaglandin dehydrogenase 15-(NAD) | | NM_001034419.2 | 5’ TGATCAGTGGAACCTACCTGG  3’ TGAGATTAGCAGCCATCGC | | | 183 | | Oliveira et al., 2015^1^ |
| *PTGES* | prostaglandin E synthase | | NM_174443.2 | 5’ GCTGCGGAAGAAGGCTTTTGCC  3’ GGGCTCTGAGGCAGCGTTCC | | | 101 | | Oliveira et al., 2015^1^ |
| *PTGES2* | prostaglandin E synthase 2 | | NM_001166554.1 | 5’ GTGGGCGGACGACTGGTTGG  3’ CGGAGGTGGTGCCTGCGTTT | | | 192 | | Oliveira et al., 2015^1^ |
| *PTGES3* | prostaglandin E synthase 3 | | NM_001007806.2 | 5’ CAGTCATGGCCAAGGTTAACAAA  3’ ATCACCACCCATGTTGTTCATC | | | 150 | | Oliveira et al., 2015^1^ |
| *PTGIS* | prostaglandin I2 (prostacyclin) synthase | | NM_174444.1 | 5’ AAGATGGGAAGCGACTGAAG  3’ ATCAGCTCCAGGTCAAACTG | | | 136 | | Oliveira et al., 2015^1^ |
| *PTGS1* | prostaglandin-endoperoxide synthase 1 | | NM_001105323.1 | 5’ CACCCGCTCATGCCCGACTC  3’ TTCCTACCCCCACCGATCCGG | | | 155 | | Oliveira et al., 2015^1^ |
| *PTGS2* | prostaglandin-endoperoxide synthase 2 | | NM_174445.2 | 5’ CCAGAGCTCTTCCTCCTGTG  3’ GGCAAAGAATGCAAACATCA | | | 161 | | Oliveira et al., 2015^1^ |
| *SLCO2A1* | solute carrier organic anion transporter family member 2A1 | | NM_174829.3 | 5’ TGTGGAGACGATGGGATTGA  3’ GGGACACGGGCCTGTCTT | | | 150 | | Oliveira et al., 2015^1^ |
| **Endogenous control** | |  | | |  |  | |  | |
| *ACTB* | actin, beta | | [NM_173979.3](http://www.ncbi.nlm.nih.gov/nucleotide/75832053?report=genbank&log$=nucltop&blast_rank=1&RID=S9KTC27B015) | 5’ GGATGAGGCTCAGAGCAAGAGA  3’ TCGTCCCAGTTGGTGACGAT | | | 76 | | Araújo et al., 2015^[[2]](#footnote-2)^ |
| *GAPDH* | glyceraldehyde-3-phosphate dehydrogenase | | NM_001034034.2 | 5’ GCCATCAATGACCCCTTCAT  3’ TGCCGTGGGTGGAATCA | | | 68 | | Araújo et al., 2015^2^ |
| *PPIA* | peptidylprolyl isomerase A | | NM_178320.2 | 5’ GCCATGGAGCGCTTTGG  3’ CCACAGTCAGCAATGGTGATCT | | | 63 | | Araújo et al., 2015^2^ |
| **Extracellular matrix assembly** | |  | | |  |  | |  | |
| *HAS3* | hyaluronan synthase 3 | | NM_001192867.1 | 5’ CTCATTGCCACGGTCATACA  3’ AGGGAGTAGAGCGACATGAA | | | 153 | | Primer Express |
| *HMMR* | hyaluronan mediated motility receptor | | NM_001206621.1 | 5’ TTGGAAAAAGAGATCCGGATTC  3’ CCCTGACGGCAGCGTTTA | | | 113 | | Primer Express |
| *HYAL1* | hyaluronoglucosaminidase 1 | | NM_001017941.1 | 5’ AGACCAAGATAGCTGCATAAG  3’ CTGTGACTGGATGCCTAAC | | | 152 | | Primer Express |
| *HYAL2* | hyaluronoglucosaminidase 2 | | NM_174347.2 | 5’ GTTGAGGTCTCCCGAAATG  3’ ACACGAAAGCTGACAAAGT | | | 126 | | Primer Express |
| **Extracellular matrix remodeling** | |  | | |  |  | |  | |
| *MMP14* | matrix metallopeptidase 14 | | NM_174390.2 | 5’ GGATTGATGCTGCTCTCTTCT  3’ CCTTCCCAGACCTTGATGTT | | | 131 | | Primer Express |
| *MMP19* | matrix metallopeptidase 19 | | NM_001075983.1 | 5’ TGCTGGGCCACTGGAGAA  3’ AGGTCAAGGGAGCCACATTG | | | 130 | | Primer Express |
| *MMP2* | matrix metallopeptidase 2 | | NM_174745.2 | 5’ CCCAGACAGTGGATGATGC  3’ TTGTCCTTCCTCCCAGGGTC | | | 159 | | Primer Express |
| *TIMP2* | TIMP metallopeptidase inhibitor 2 | | NM_174472.4 | 5’ TGCAGACATAGTGATCAGGGCCA  3’ AATCCGCTTGATGGGGTTGCCG | | | 88 | | Primer Express |
| *TIMP3* | TIMP metallopeptidase inhibitor 3 | | NM_174473.4 | 5’ CCTTTGGCACGATGGTCTACA  3’ CTCGGCCTGTCAGCAGGTA | | | 154 | | Primer Express |
| **Growth factor signaling** | |  | | |  |  | |  | |
| *EDN3* | endothelin 3 | | NM_001101979.1 | 5’ GTGTTAGCCTTGACCAAATGC  3’ GGAGTTGATGTAGAGACCAGTTT | | | 157 | | Primer Express |
| *EGFR* | epidermal growth factor receptor | | XM_592211.8 | 5’ ATGCTCTATGACCCTACCAC  3’ TTCCGTTACAAACTTTGCCA | | | 132 | | Primer Express |
| *FGF2* | estrogen receptor 1 | | NM_174056.4 | 5’ AGCACTGGCACTACTACA  3’ AGCCAACTCCTAACATCCTA | | | 141 | | Primer Express |
| *FGFR2* | fibroblast growth factor receptor 2 | | NM_001205310.1 | 5’ TTGACGTTGTTGAGCGATCAC  3’ GGCTGGGCATCGCTGTAC | | | 119 | | Primer Express |
| *FLT1* | fms related tyrosine kinase 1 | | NM_001191132.3 | 5’ CCGAAAACTGAAAAGGTCGTCTT  3’ ACTTCATCCGGGTCCATGATAA | | | 146 | | Primer Express |
| *GRB7* | growth factor receptor bound protein 7 | | NM_001046014.1 | 5’ TGCCCCCATGTCATAAAGGT  3’ CCCCCAGTTCTCGTCACTCA | | | 133 | | Primer Express |
| *IGF1* | insulin like growth factor 1 | | NM_001077828.1 | 5’ CATCCTCCTCGCATCTCTTC  3’ CTCCAGCCTCCTCAGATCAC | | | 239 | | Primer Express |
| *IGF1R* | insulin like growth factor 1 receptor | | NM_001244612.1 | 5’ AGAGACATCTATGAGACGGAC  3’ CAGCTCAAACAGCATGTCAG | | | 261 | | Primer Express |
| *IGF2* | insulin like growth factor 2 | | NM_174087.3 | 5’ GACCGCGGCTTCTACTTCAG  3’ AAGAACTTGCCCACGGGGTAT | | | 203 | | Primer Express |
| *IGF2R* | insulin like growth factor 2 receptor | | NM_174352.2 | 5’ AGAAAAGCGTGCACGTGCACTTGTC  3’ CGCCTACAGCGAGAAGGGCTTAGTCC | | | 293 | | Primer Express |
| *IGFBP7* | insulin like growth factor binding protein 7 | | NM_001102300.2 | 5’ AAGGAAGATGCCGGAGAATATG  3’ TTACAGCTCAGCACCTTCAC | | | 130 | | Primer Express |
| *KDR* | kinase insert domain receptor | | NM_001110000.1 | 5’ AGACCGGCTGAAACTAGGTAAGC  3’ CGTTGAGATGGTGGCCAATA | | | 198 | | Primer Express |
| **Interferon Signaling** | |  | | |  |  | |  | |
| *IFI6* | interferon, alpha-inducible protein 6 | | NM_001075588.1 | 5’ GGCGGTATCGCTCTTCCTATG  3’ GCTCGAGTCGCTGTTTTCCT | | | 98 | | Primer Express |
| *IFNAR2* | interferon (alpha, beta and omega) receptor 2 | | NM_174553.2 | 5’ CTGGTCATTTGTATGGGCTCTTT  3’ GTATCCCGGGACTGTCGAATT | | | 128 | | Primer Express |
| *IRF6* | interferon regulatory factor 6 | | NM_001076934.1 | 5’ GGTCTGCTCCTTGGGATGAG  3’ ATGGGAGAACCATTGATGTTCAG | | | 128 | | Primer Express |
| *ISG15* | ISG15 ubiquitin-like modifier | | NM_174366.1 | 5’ AGAGAGCCTGGCACCAGAAC  3’ TTCTGGGCGATGAACTGCTT | | | 130 | | Primer Express |
| *MX1* | MX dynamin-like GTPase 1 | | NM_173940.2 | 5’ AGACGAGTGGAAAGGCAAAGTC  3’ GATGGCAATCTGGGCTTCAC | | | 98 | | Primer Express |
| *MX2* | MX dynamin-like GTPase 2 | | NM_173941.2 | 5’ TCAGAGACGCCTCAGTCGAA  3’ ACGTTTGCTGGTTTCCATGAA | | | 109 | | Primer Express |
| *OAS1Y* | 2',5'-oligoadenylate synthetase 1 | | NM_001040606.1 | 5’ TAGGCCTGGAACATCAGGTC  3’ TTTGGTCTGGCTGGATTACC | | | 104 | | Primer Express |
| **Oxidative Stress** | |  | | |  |  | |  | |
| *CAT* | catalase | | NM_001035386.2 | 5’ CGCGCAGAAACCTGATGTC  3’ GGAATTCTCTCCCGGTCAAAG | | | 150 | | Ramos et al., 2015^[[3]](#footnote-3)^ |
| *GPX4* | glutathione peroxidase 4 | | NM_174770.3 | 5’ TCACCAAGTTCCTCATTGACAAGA  3’ TTCTCGGAACACAGGCAACA | | | 150 | | Ramos et al., 2015^3^ |
| *SOD1* | superoxide dismutase 1, soluble | | NM_174615.2 | 5’ GTTGGAGACCTGGGCAATGT  3’ TCCACCCTCGCCCAAGTCAT | | | 151 | | Ramos et al., 2015^3^ |
| *SOD2* | superoxide dismutase 2, soluble | | NM_201527.2 | 5’ CCCATGAAGCCTTTCTAATCCTG  3’ TTCAGAGGCGCTACTATTTCCTTC | | | 307 | | Ramos et al., 2015^3^ |
| **Polyamine Regulation and proteolysis** | |  | | |  |  | |  | |
| *AMD1* | adenosylmethionine decarboxylase 1 | | NM_173990.2 | 5’ TGCTGGAGGTTTGGTTCTC  3’ TCAAAAGTATGTCCCACTCGG | | | 96 | | -Ramos et al., 2014^[[4]](#footnote-4)^ |
| *ODC1* | ornithine decarboxylase 1 | | NM_174130.2 | 5’ GTGAACCATGGAGTATATGGGTC  3’ CTCATCTGGTTTGGGTCTCTTC | | | 93 | | Ramos et al., 2014^4^ |
| *ANPEP* | alanyl aminopeptidase, membrane | | NM_001075144.1 | 5’ ATCCGGATGCTCTCGAATTTC  3’ TCTGATAGGCAAAGGTCTGCAA | | | 82 | | Primer Express |
| *EED* | embryonic ectoderm development | | NM_001040494.2 | 5’ GAAATCCGGTTGTTGCAGTCTT  3’ TGGCCAACATAGTGCTTTATGC | | | 174 | | Primer Express |
| **Secretory activity** | |  | | |  |  | |  | |
| *GRP* | gastrin-releasing peptide | | NM_001101239.1 | 5’ GTGGGAAGAAGCGACAAGGA  3’ TGCTGAGGACCTGTGTCTTTGA | | | 148 | | Primer Express |
| *LTF* | lactotransferrin | | NM_180998.2 | 5’ CGTGGCAGTTGTCAAGAA  3’ GCACAGCTCTGACTAAAGAA | | | 169 | | Primer Express |
| *MCOLN3* | mucolipin 3 | | NM_001192367.1 | 5’ ACCAGCATACATCTCCCTCT  3’ TGGCAAGTTTCCAGGGTTT | | | 124 | | Primer Express |
| *PIP* | prolactin-induced protein | | NM_001080913.1 | 5’ GCTGCCCTGCTTCTGATTCT  3’ CCACGGTGGCCTCTTCACT | | | 128 | | Primer Express |
| *RBP4* | retinol binding protein 4 | | NM_001040475.2 | 5’ ACCTGCGCTGACAGCTACTCTT  3’ CAGTAACCGTTGTGAGGGATCA | | | 138 | | Primer Express |
| *SCAMP1* | secretory carrier membrane protein 1 | | NM_001076054.2 | 5’ ACCCTTTCAAGGACCCATCAG  3’ CAAGGCATGTTCCTTTGCAA | | | 198 | | Primer Express |
| *SCAMP2* | secretory carrier membrane protein 2 | | NM_001102170.1 | 5’ CATGTCGTCCTTTGACACCAA  3’ TCGCTGCATTTGTCTCTGAGA | | | 132 | | Primer Express |
| *SCAMP3* | secretory carrier membrane protein 3 | | NM_001035426.1 | 5’ TGAAGCGGATCCACTCTTTGT  3’ GCCCGGAAGGCATTTTCT | | | 146 | | Primer Express |
| *SERPINA14* | serpin peptidase inhibitor, clade A (alpha-1 antiproteinase, antitrypsin), member 14 | | NM_174797.3 | 5’ ATATCATCTTCTCCCCCATGG  3’ GTGCACATCCAACAGTTTGG | | | 123 | | Araújo et al., 2015^[[5]](#footnote-5)^2 |
| *SPP1* | secreted phosphoprotein 1 | | NM_174187.2 | 5’ TCCGCCCTTCCAGTTAAACC  3’ TGTGGTGTTAGGAAAGTCTGCT | | | 131 | | Primer Express |
| **Sex steroid signaling** | |  | | |  |  | |  | |
| *ESR1* | estrogen receptor 1 | | NM_001001443.1 | 5’ CAGGCACATGAGCAACAAAG  3’ TCCAGCAGCAGGTCGTAGAG | | | 82 | | Primer Express |
| *ESR2* | estrogen receptor 2 | | NM_174051.3 | 5’ GTAGAGAGCCGCCATGAATAC  3’ CAATGGATGGCTAAAGGAGAGA | | | 159 | | Primer Express |
| *GPER* | G protein-coupled estrogen receptor 1 | | XM_015469468.1 | 5’ CCTGTACACCATCTTCCTCTTC  3’ CGATGTCATAGTACTGCTCGTC | | | 189 | | Primer Express |
| *OXTR* | oxytocin receptor | | NM_174134.2 | 5’ AAGATGACCTTCATCGTCGTG  3’ CGTGAAGAGCATGTAGATCCAG | | | 175 | | Primer Express |
| *PAQR8* | progestin and adipoQ receptor family member VIII | | NM_001101135.1 | 5’ TGCCCCTGCTCGTCTATGTC  3’ CCCACGTAGTCCACGAAGTAGAA | | | 121 | | Primer Express |
| *PGR1* | progesterone receptor isoform A | | NM_001205356.1 | 5’ ACTACCTGAGGCCGGATT  3’ CCCTTCCATTGCCCTCTTAAA | | | 163 | | Primer Express |
| *PGRMC1* | progesterone receptor membrane component 1 | | NM_001075133.1 | 5’ AGGGGCCGTATGGAGTCTTT  3’ CCACATGATGGTACTTGAAAGTGAA | | | 172 | | Primer Express |
| *PGRMC2* | progesterone receptor membrane component 2 | | NM_001099060.1 | 5’ CAGGGGAAGAACCGTCAGAA  3’ ATGAAGCCCCACCAGACATT | | | 282 | | Primer Express |
| **Solute and water transport** | |  | | |  |  | |  | |
| *AQP1* | aquaporin 1 | | NM_174702.3 | 5’ AACCCTGCCCGGTCCTT  3’ CGCGGTCTGTGAGGTCACT | | | 149 | | Primer Express |
| *AQP4* | aquaporin 4 | | NM_181003.3 | 5’ GTGTCTGTTGCAGTGAGAT  3’ CAAAGGGACCTGGGATTTAG | | | 157 | | Primer Express |
| *CLDN10* | claudin 10 | | NM_001014857.1 | 5’ AGCCTCACTCTGCCTAAT  3’ TTCTCTGCCGTGATACTTTG | | | 134 | | Primer Express |
| *SLC13A5* | solute carrier family 13, member 5 | | NM_001191446.1 | 5’ GGAAGCAGATGGAGCCTTT  3’ ATCATGGAGGCAAAGATGGG | | | 137 | | Primer Express |
| *SLC1A4* | solute carrier family 1, member 4 | | NM_001081577.1 | 5’ ATCTTGATAGGCGTGGTTTC  3’ GCAACACTGGTTCTCTCTATAA | | | 132 | | França et al., 2015^[[6]](#footnote-6)^ |
| *SLC2A1* | solute carrier family 2, member 1 | | NM_174602.2 | 5’ ATCATCTTCACCGTGCTCCTGGTT  3’ TGTCACTTTGACTTGCTCCTCCC | | | 127 | | França et al., 2015^5^ |
| *SLC5A6* | solute carrier family 5, member 6 | | NM_001046219.2 | 5’ TCCCTCAGCACCATATCCTC  3’ CCAAGGCAGAAGAGTCCAAG | | | 248 | | Primer Express |
| *SLC7A8* | solute carrier family 7, member 8 | | NM_001192889.2 | 5’ GAGATTGGATTGGTCAGTGG  3’ CTCCCACAACTGTGATAAG | | | 156 | | Primer Express |

1. Oliveira ML, D'Alexandri FL, Pugliesi G, Van Hoeck V, Mesquita FS, Membrive CMB, et al. Peri-ovulatory endocrine regulation of the prostanoid pathways in the bovine uterus at early dioestrus. Reproduction, Fertility and Development. 2015. [↑](#footnote-ref-1)
2. 1 Oliveira ML, D'Alexandri FL, Pugliesi G, Van Hoeck V, Mesquita FS, Membrive CMB, et al. Peri-ovulatory endocrine regulation of the prostanoid pathways in the bovine uterus at early dioestrus. Reproduction, Fertility and Development. 2015.

   Araújo ER, Sponchiado M, Pugliesi G, Van Hoeck V, Mesquita FS, Membrive CMB, et al. Spatio-specific regulation of endocrine-responsive gene transcription by periovulatory endocrine profiles in the bovine reproductive tract. Reproduction, Fertility and Development. 2015. [↑](#footnote-ref-2)
3. Ramos RS, Oliveira ML, Izaguirry AP, Vargas LM, Soares MB, Mesquita FS, et al. The periovulatory endocrine milieu affects the uterine redox environment in beef cows. Reproductive Biology and Endocrinology. 2015;13(1):39. [↑](#footnote-ref-3)
4. Ramos RdS, Mesquita FS, D'Alexandri FL, Gonella‐Diaza AM, Papa PdC, Binelli M. Regulation of the polyamine metabolic pathway in the endometrium of cows during early diestrus. Molecular reproduction and development. 2014;81(7):584-94. [↑](#footnote-ref-4)
5. 2 Araújo ER, Sponchiado M, Pugliesi G, Van Hoeck V, Mesquita FS, Membrive CMB, et al. Spatio-specific regulation of endocrine-responsive gene transcription by periovulatory endocrine profiles in the bovine reproductive tract. Reproduction, Fertility and Development. 2015. [↑](#footnote-ref-5)
6. França MR, Mesquita FS, Lopes E, Pugliesi G, Van Hoeck V, Chiaratti MR, et al. Modulation of periovulatory endocrine profiles in beef cows: consequences for endometrial glucose transporters and uterine fluid glucose levels. Domestic animal endocrinology. 2015;50:83-90. [↑](#footnote-ref-6)
