# Supplementary material for: Pre-hatching embryo-dependent and -independent programming of endometrial function in cattle
Source: PLoS One. 2017 Apr 19;12(4):e0175954. doi: 10.1371/journal.pone.0175954 (PMC5397052; doi:10.1371/journal.pone.0175954)
Supplement: S2 Table — (DOCX) [file pone.0175954.s002.docx]

| **Gene** | **Overall effects** | | | | | |  | **Inter-regions Comparisons** | | | | | | | | | | | | | |
| --- | --- | --- | --- | --- | --- | --- | --- | --- | --- | --- | --- | --- | --- | --- | --- | --- | --- | --- | --- | --- | --- |
|  | **Group** | **Region** | | | **Group*Region** | |  | **UTJ *vs.* IA** | **UTJ *vs.* IM** | | | **UTJ *vs.* IP** | | | **IA *vs.* IM** | | | **IA *vs.* IP** | | | **IM *vs.* IP** |
| **Cell-cell adhesion** | | | | | | |  |  |  | | |  | | |  | | |  | | |  |
| *FN1* | ns | ** | | | ns | |  | ** | ** | | | ** | | | ns | | | ns | | | ns |
| *ICAM1* | ns | ** | | | ns | |  | ** | ** | | | ** | | | ** | | | ** | | | ˟ |
| *ICAM3* | ns | ** | | | ns | |  | ** | ** | | | ** | | | ns | | | ˟ | | | ns |
| *ITFG3* | ns | ** | | | ns | |  | ** | ** | | | ** | | | ns | | | * | | | ns |
| *LGALS1* | ns | * | | | ns | |  | ns | ns | | | ns | | | ** | | | * | | | ns |
| *LGALS7B* | ns | * | | | ns | |  | * | ** | | | * | | | * | | | ns | | | ns |
| *LGALS9* | ns | * | | | ns | |  | ns | ** | | | ** | | | ns | | | ns | | | ns |
| *MUC1* | ns | ˟ | | | ns | |  | * | * | | | * | | | ˟ | | | * | | | ns |
| *VIL1* | ns | ns | | | ns | |  | ns | ns | | | ns | | | ns | | | ns | | | ns |
| **Eicosanoid metabolic process** | | | | | | |  |  |  | | |  | | |  | | |  | | |  |
| *AKR1B1* | ns | ** | | | ns | |  | ** | ** | | | ** | | | * | | | ns | | | ns |
| *PTGES2* | ns | ** | | | ns | |  | ** | ** | | | ** | | | ns | | | ˟ | | | ns |
| *PTGES3* | ns | ** | | | ns | |  | ** | ** | | | ˟ | | | ns | | | ˟ | | | ns |
| *PTGIS* | ns | ** | | | ns | |  | ** | ** | | | ** | | | ˟ | | | ˟ | | | ns |
| *PTGS1* | ns | ** | | | ns | |  | ** | ** | | | ** | | | ns | | | ns | | | ns |
| *PTGS2* | ns | ** | | | ns | |  | ** | ˟ | | | ** | | | ** | | | ns | | | * |
| *SLCO2A1* | ns | ** | | | ns | |  | ** | ** | | | ** | | | ns | | | ns | | | ns |
| **Extracellular matrix assembly** | | | | | | |  | | |  | | |  | | |  | | |  | | |
| *HAS3* | ns | ** | | | ns | |  | ** | ** | | | ** | | | ns | | | * | | | ns |
| *HMMR* | ns | ** | | | ns | |  | ** | ** | | | ** | | | ns | | | ns | | | ns |
| *HYAL1* | ns | ** | | | ns | |  | ** | ** | | | ** | | | ns | | | ns | | | ns |
| *HYAL2* | ns | ** | | | ns | |  | ** | ** | | | ** | | | ns | | | ns | | | ns |
| **Extracellular matrix remodeling** | | | | | | |  | | |  | | |  | | |  | | |  | | |
| *MMP14* | ns | ** | | | ns | |  | ** | ** | | | ** | | | ns | | | ns | | | ns |
| *MMP19* | ns | ** | | | ns | |  | ** | ** | | | ** | | | ˟ | | | ns | | | ns |
| *MMP2* | ns | ** | | | ns | |  | ** | ** | | | ** | | | ˟ | | | ns | | | ns |
| *TIMP2* | ns | ** | | | ns | |  | ** | ** | | | ** | | | ns | | | ˟ | | | * |
| *TIMP3* | ns | ** | | | ns | |  | ** | ** | | | ** | | | ns | | | ns | | | ns |
| **Growth factor signaling** | | | | | | |  | | |  | | |  | | |  | | |  | | |
| *EDN3* | ns | ** | | | ns | |  | ** | ** | | | ** | | | * | | | ** | | | ns |
| *EGFR* | ns | * | | | ns | |  | * | ** | | | * | | | ns | | | ns | | | ns |
| *FGF2* | ns | * | | | ns | |  | ** | * | | | ** | | | ns | | | ns | | | ns |
| *FGFR2* | ns | ** | | | ns | |  | ** | ** | | | ** | | | * | | | ˟ | | | ns |
| *FLT1* | ns | | ** | | ns | |  | ** | ** | | | ** | | | ** | | | ns | | | ns |
| *GRB7* | ns | | ** | | ns | |  | ** | ** | | | ** | | | ** | | | ** | | | ns |
| *IGF1* | ns | | ** | | ns | |  | ** | ** | | | ** | | | ns | | | ns | | | ns |
| *IGF1R* | ns | | ** | | ns | |  | ** | ** | | | ** | | | ns | | | ns | | | ns |
| *IGF2* | ns | | ** | | ns | |  | * | ** | | | ** | | | ˟ | | | ns | | | ns |
| *IGF2R* | ns | | * | | ns | |  | ** | * | | | ** | | | ns | | | ns | | | ns |
| *IGFBP7* | ns | | ˟ | | ns | |  | ns | * | | | * | | | * | | | ns | | | ns |
| *KDR* | ns | | ** | | ns | |  | ** | ** | | | ** | | | ** | | | * | | | ns |
| **Interferon Signaling** | | | | | | |  | | |  | | |  | | |  | | |  | | |
| *IFI6* | ns | | ** | | ns | |  | ** | ** | | | ** | | | ns | | | ns | | | ns |
| *IFNAR2* | ns | | * | | ns | |  | * | * | | | * | | | ns | | | ns | | | ns |
| *IRF6* | ns | | ** | | ns | |  | ** | ** | | | ** | | | ns | | | ns | | | ns |
| **Oxidative Stress** | | | |  | |  | |  | | |  | | |  | | |  | | |  | |
| *CAT* | ns | | ** | | ns | |  | ** | ** | | | * | | | ns | | | ns | | | ns |
| *GPX4* | ns | | ** | | ns | |  | ** | ** | | | ** | | | ns | | | ns | | | ns |
| *SOD1* | ns | | ** | | ns | |  | ** | ** | | | ** | | | ns | | | ns | | | ns |
| *SOD2* | ns | | ns | | ns | |  | ns | ns | | | ns | | | ns | | | ns | | | ns |
| **Polyamine Regulation and proteolysis** | | | | | | |  |  |  | | |  | | |  | | |  | | |  |

**S2 Table.** **Summary of regional effects in the transcripts abundance measured by Real Time PCR in the uterotubal junction (UTJ), anterior (IA), medial (IM) and posterior (IP) samples of the ipsilateral uterine horn.** (Continued)

| *ODC1* | ns | ** | ns |  | ** | ** | ** | ns | ns | ns |
| --- | --- | --- | --- | --- | --- | --- | --- | --- | --- | --- |
| *ANPEP* | ns | ** | ns |  | ** | ** | ** | ns | ns | ns |
| *EED* | ns | ** | ns |  | ns | ns | ns | ns | ns | ns |
| **Secretory activity** | | | |  |  |  |  |  |  |  |
| *LTF* | ns | ns | ns |  | ns | ns | ns | ns | ns | ns |
| *MCOLN3* | ns | ** | ns |  | ** | ** | ** | ns | * | ns |
| *PIP* | ns | ns | ns |  | ns | ns | ns | ns | ns | ns |
| *RBP4* | ns | ** | ns |  | ** | ** | ** | ** | * | ns |
| *SCAMP1* | ns | * | ns |  | ** | * | ns | ns | ns | ns |
| *SCAMP2* | ns | ** | ns |  | ** | ** | ** | * | ** | ns |
| *SCAMP3* | ns | ** | ns |  | ** | ** | ** | ˟ | ns | ns |
| *SERPINA14* | ns | * | ns |  | ** | * | ns | ns | ns | ns |
| *SPP1* | ns | * | ns |  | * | ns | ns | ˟ | ˟ | ns |
| **Sex steroid signaling** | | | |  |  |  |  |  |  |  |
| *ESR1* | ns | ** | ns |  | ** | ** | ** | ns | ns | ns |
| *ESR2* | ns | ** | ns |  | ** | ** | ** | ** | ** | ˟ |
| *GPER* | ns | ˟ | ns |  | * | * | * | ns | ns | ˟ |
| *OXTR* | ns | ** | ns |  | ** | ** | ** | * | * | ˟ |
| *PAQR8* | ns | ** | ns |  | ** | ** | ** | ns | * | ns |
| *PGR1* | ns | ˟ | ns |  | ˟ | ˟ | ˟ | ns | ns | ns |
| *PGRMC1* | ns | ** | ns |  | ** | ** | ** | ns | ns | ns |
| *PGRMC2* | ns | ns | ns |  | ns | ns | ns | ns | ns | ns |
| **Solute and water transport** | | | |  |  |  |  |  |  |  |
| *AQP1* | ns | ns | ns |  | ns | ns | ns | ns | ns | ns |
| *AQP4* | ns | ** | * |  | ** | ns | ns | * | * | ns |
| *CLDN10* | ns | ˟ | ns |  | ns | ns | ns | ns | ns | * |
| *SLC13A5* | ns | ** | ns |  | ** | ** | ** | ns | ns | ns |
| *SLC1A4* | ns | ns | ˟ |  | ns | ns | ˟ | ns | ns | ns |
| *SLC2A1* | ns | ** | ns |  | ** | ** | ** | * | ** | ns |
| *SLC5A6* | ns | ** | ns |  | ** | ** | ** | ns | ns | ns |
| *SLC7A8* | ns | ** | ns |  | ** | ** | * | ns | ns | ns |

Magnitude of effect is indicated by: ***P* ≤ 0.01; **P* ≤ 0.05; ˟*P* ≤ 0.1; ns: not significant (*P* > 0.1).
