# Supplementary figures and images for: Pre-hatching embryo-dependent and -independent programming of endometrial function in cattle
Source: PLoS One. 2017 Apr 19;12(4):e0175954. doi: 10.1371/journal.pone.0175954 (PMC5397052; doi:10.1371/journal.pone.0175954)

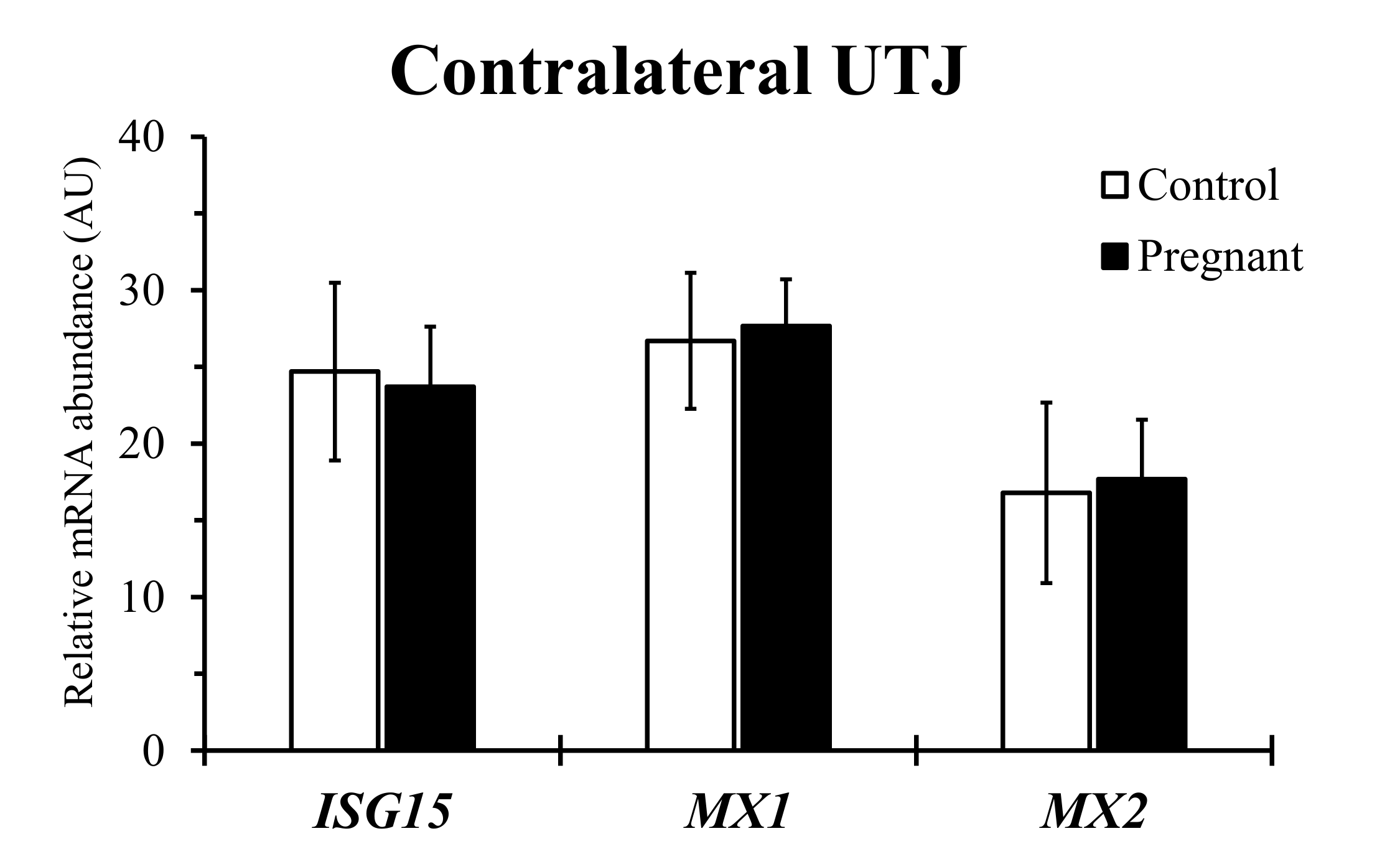

Supplement: S1 Fig — No significant mean differences were detected (P > 0.1). (TIF) [file pone.0175954.s003.tif]
